# Abstracts of the 14th C1-inhibitor Deficiency and Angioedema Workshop: part 2

**DOI:** 10.1186/s13223-026-01017-1

**Published:** 2026-10-20

## O-11

### Genetic double strike in C1-INH deficiency hereditary angioedema: when rare become ultra rare—Patient case series from a monocentric Italian cohort

Paola Triggianese^1^, Enrica Marchionni^2^, Liliana Mannucci^2^, Francesca Cedola^1^, Federica Carla Sangiuolo^1,2^, Mauro Cancian^3^, Giuseppe Novelli^1,2^

^1^Department of Biomedicine and Prevention, University of Rome Tor Vergata, Rome, Italy; ^2^Medical Genetics Unit, Policlinico Tor Vergata, University of Rome Tor Vergata, Rome, Italy; ^3^Department of Systems Medicine, University Hospital of Padua, Padua, Italy

Allergy, Asthma & Clinical Immunology 2026, 22(Suppl 1):O-11

**Background:** Hereditary angioedema (MIM#106,100) is a rare autosomal dominant disease characterized by recurrent cutaneous-submucosal edema. Heterozygous pathogenic variants in *SERPING1* (MIM*606,860) coding for C1-esterase inhibitor (C1-INH) cause C1-INH-HAE (type 1–2).

We reported four C1-INH-HAE cases in which the combination of clinically driven design process and genomic approaches disclosed the coexistence of two rare genetic diseases.

**Methods:** Affected Patients were women in 75% of cases: a positive C1-INH-HAE family history occurred in 3 patients (66.7% type 1) while one case was sporadic (type 1). Patients were Caucasian and referred to the ITACA Reference Center in Rome (Policlinico Tor Vergata), Italy. In all of them, the genetic identification of a concurrent disease occurred after the C1-INH-HAE diagnosis. It was documented by clinically driven design processes in 75% (2 F, 1 M) of cases while in the sporadic case (1 F) it was an incidental finding. Three of the four documented diseases were rare neurological disorders.

Case 1 (M) presented HAE symptoms onset in pediatric age: he inherited the *SERPING1* pathogenic variant from his affected mother. The Patient presented progressive loss of coordination and balance and hypertrophic cardiomyopathy: autosomal recessive Friedreich Ataxia (MIM#229,300) was diagnosed in pediatric age (6 y.o.), due to a compound heterozygous GAA expansion in *FXN* gene.

Case 2 (F) presented HAE symptoms in puberty: she inherited the *SERPING1* pathogenic variant from her affected father. At stage II Tanner, she showed generalized acute epilepsy with drug-resistant myoclonic crisis: Myoclonic Epilepsy of Lafora 2 (MIM#620,681) was diagnosed due to compound heterozygous *NHLRC1* pathogenic variants.

Case 3 (F) was a sporadic C1-INH-HAE case in which molecular diagnosis was made at 55 y.o. She presented a very rare deletion of the whole *SERPING1* gene and neuropathic symptoms compatible with the incidentally disclosed canonical *PMP22* deletion causing Hereditary Neuropathy with liability to Pressure Palsies (HNPP, MIM#162,500).

Case 4 (F) was born with congenital cardiopathy and mild dysmorphisms: Noonan Syndrome (MIM#163,950) was diagnosed, detecting a de novo* PTPN11* pathogenic variant. At the same stage I Tanner, she also presented abdominal attacks: inherited *SERPING1* pathogenic variant from her affected father was thus documented.

**Conclusions**: We described the first Italian case series from C1-INH-HAE patients with *SERPING1* pathogenic variants in whom genetic analyses detected the simultaneous presence of rare conditions that co-occurred in unrelated patients. Although rare, the possibility of being affected by two inherited genetic conditions should be suspected when clinical phenotypes are incoherent with primary diagnoses. Consent to publish had been obtained.

## O-12

### Cosegregation study of kallikrein-kinin system genes in a cohort of 8 unrelated families with HAE-C1-INH in Southern Spain

Krasimira Baynova^1^, Teresa De Aramburu Mera^1^, José Manuel Lucena^2^, Raúl García^2^, Stefan Cimbollek^1^

^1^Allergy Department, Spanish National Center for Angioedema, Virgen del Rocío University Hospital, Seville, Spain; ^2^Immunology Department, Spanish National Center for Angioedema, Virgen del Rocío University Hospital, Seville, Spain

Allergy, Asthma & Clinical Immunology 2026, 22(Suppl 1):O-12

**Background:** HAE-C1-INH is clinically highly variable from one patient to another, within the same patient and among relatives carrying the same variant in the SERPING1 gene.

Our objective was to study low prevalence variants of genes related to the kallikrein-kinin system (KKS) and analyze their association with different clinical phenotypes in patients with HAE-C1-INH.

**Materials and methods:** 8 unrelated families with HAE-C1-INH were selected (39 patients, 16 males). Clinical data was obtained for patients over 18 years, with a different phenotypes, and at least 3 subjects per family were evaluated. The severity of the disease was established as mild, moderate, severe and very severe phenotype. Patients were informed about the genetic testing and signed informed consent. For each patient, DNA form peripherial blood sample was studied using next-generation sequencing platform targeting the 45 genes from KKS.

**Results:** 7 families presented HAE-C1-INH type 1 and 1 family-type 2. Different SERPING1 casual variants in heterozygosity were found for each family. Variants in the tested KKS related genes were found as follows:

**Family 1:** The CPN1 c.533G > A variant was present in heterocygosity in all patients with mild phenotype and was negative in the patients with moderate and severe phenotype.

**Family 2:** SERPINF1 variant c.395C > G was found in 6 members but no correlation with phenotype was established.

**Family 3:** Variants MASP2 c.890C > T and PLG c.1567C > T were detected with no correlation with phenotype.

**Family 4:** Variants KRT1 c.1360G > T, SERPINF1 c.395C > G, TLR4 c.1610C > T were detected in 3 very severe patients but were negative in the mildest case of the family and in his sibling with very severe phenotype.

**Family 5:** Variants MASP2 c.172C > T, MME c.22A > G, IL5 c.146C > T, CPN1 c.503C > T, ANGPT1 c.1151G > A, ELANE c.770C > T, XPNPEP2 c.1865G > A were detected with no correlation with phenotype.

**Family 6**: Variants MASP1 c.1277G > A, ACE c.1060G > A, C1R c.887G > A were found without correlation with phenotype.

**Family 7:** Variant CPN1 c.533G > A was found in heterocygosity in one of the mildest patients but not found in the two most severe patients of the family.

**Family 8:** Variants C1R c.887G > A, ACE c.1060G > A, MASP1 c, 1277G > A were found without correlation with phenotype.

**Conclusions:** We have identified the CPN1 variant c.533G > A whith protective role in two of the studied families. All other detected variants in KKS genes were not associated to specific phenotype in our cohort of HAE-C1-INH patients. More studies are needed in the field of gentoype-phenotype association in HAE.

## O-13

### Identification of a novel XPNPEP2 variant associated with hereditary angioedema with normal C1-INH activity: A molecular insight

Sandra Nieto*, Rolando Laurel Laurel.

Asociación Mexicana de Angioedema Hereditario, Mexico City, Mexico

*sandranietoaeh@gmail.com

Allergy, Asthma & Clinical Immunology 2026, 22(Suppl 1):O-13

**Background:** Hereditary angioedema (HAE) is an autosomal dominant disease characterized by recurrent episodes of subcutaneous or mucosal angioedema, caused by a quantitative or qualitative deficiency of C1 esterase inhibitor (C1-INH). It has high morbidity and mortality rates, potentially fatal in 25–40% of cases. In Mexico, there is an estimated delay in diagnosis until the age of 22. Clinical manifestations can occur at any anatomical site and present a differential diagnosis for a wide range of conditions. The diagnosis is based on clinical findings and, when available, family history [1]. Hereditary angioedema (HAE) is usually caused by mutations in the C1-INH gene (SERPING1). However, other genetic variants have been identified as the underlying causes of this disorder, including mutations in the KNG1, plasminogen, myoferlin, HS3ST6, factor XII, and angiopoietin-1 genes. These mutations lead to a dysregulated bradykinin pathway, resulting in recurrent episodes of angioedema [2].

**Case Report:** A 63-year-old patient has suffered from this condition since the mid-1990s. In March 2000, he visited the McGovern Clinic in Houston, where he was diagnosed with Angioedema. Despite treatment with immunotherapy due to suspicion of multiple allergies, symptoms have persisted up to the present day. Typically, the patient experiences swelling in various parts of the face (eyelids, lips), often accompanied by tiredness and mild headaches. In recent years, he has also experienced a general sense or hunch that an attack is imminent, a sensation that often precedes the episodes. Additionally, he has had urticaria on his arms and legs a couple of times.

Lack of sleep and fatigue have been identified as triggers for some attacks, and he experiences 2 to 4 attacks per month since 2020. Laboratory results show normal complement levels and normal C1-inhibitor levels. Given the high suspicion of HAE with normal C1-INH, genetic studies were performed in July 2023, revealing the variant XPNPEP2 NM_003399.5:c.373A > T p. (Met125Leu) hemizygous. This is the first case of hereditary angioedema (HAE) in his family.

**Conclusion:** This case highlights the identification of a novel hemizygous variant in the XPNPEP2 gene (NM_003399.5:c.373A > T p.(Met125Leu)) as a likely cause of hereditary angioedema (HAE) with normal C1-INH activity. The clinical presentation of recurrent angioedema episodes, with typical triggers and prodromal symptoms, supports this genetic finding. The discovery of this variant expands the molecular understanding of HAE, especially in cases with normal C1-INH levels, offering new insights for diagnosis and potential therapeutic approaches in such rare cases. Further research into this variant's role in HAE pathophysiology is warranted. Consent to publish had been obtained.

References:

[1]Sinnathamby ES. Hereditary Angioedema: Diagnosis, Clinical Implications, and Pathophysiology. Adv Ther. 2023

[2]Germenis AE, Speletas M. Genetics of Hereditary Angioedema Revisited. Clin Rev Allergy Immunol. 2016 Oct;51(2):170-82. https://doi.org/10.1007/s12016-016-8543-x. PMID: 27116602.

## O-14

### Preliminary results from epidemiological survey of hereditary angioedema in India—just a tip of the iceberg

Ankur Kumar Jindal*, Samipa Samir Mukherjee, Suprit Basu, Prabal Barman, Archan Sil, Sagar Bhattad, G. Anjani, Yasmeen Bhatt, Ram Gulati, Himanshi Chaudhary, Avinash Sharma, N. Johnson, Pratap Patra, Debasis Patro, Sendhil M. Kumaran, Sunil Dogra

Postgraduate Institute of Medical Education and Research, Chandigarh, India

Allergy, Asthma & Clinical Immunology 2026, 22(Suppl 1):O-14

*On behalf of the ‘Task force Epidemiological Survey HAE India’ from the Hereditary Angioedema Society of India (HAESI).

**Background:** The global prevalence of HAE has been reported to be varying from 1:10,000 to 1:150,000. However, there are no epidemiological data on HAE from developing countries including India. It has been estimated that there are at least 30,000 patients with HAE in India based on extrapolation from global prevalence. Therefore, in this study we carried out an epidemiological survey to understand the prevalence of HAE patients in India and prevalent practices for diagnosis and management of HAE amongst health care providers in the country.

**Patients and Methods:** A task force of physicians, who also are members of the HAE society of India (www.haesi.in), was formed in June 2024 and a Google form-based questionnaire. Questionnaire was circulated amongst physicians belonging to Dermatology, Pediatrics, Otolaryngorhinology, Allergy, Immunology, Rheumatology and Gastroenterology specialities. The responses were collected between July 2024 to November 2024 and the data were analysed.

**Results:** We received a total of 161 responses and the total number of diagnosed patients as reported in the survey were 722 (HAEC1-INH-Type 1: 506; HAEC1-INH-Type 2: 67, nlC1-INH-HAE: 26, not sure: 123). Of the total responses, 84% reported suspecting a diagnosis of HAE in a patient and 57% reported diagnosing a patient with HAE. Approximately 91% physicians confirmed the diagnosis of HAE using laboratory investigations while 9% diagnosed them clinically only. When asked about how patients were screened, most physicians (68%) used C4 and C1-inhibitor levels to screen HAE while only a small proportion used C1-inhibitor function to screen patients with HAE. Large majority of physicians reported having diagnosed < 5 patients. The most common speciality managing patients with HAE were Dermatology followed by Pediatrics, Rheumatology, Clinical Immunology and Rheumatology. Of the treatment options used by the physicians for on demand treatment use of fresh frozen plasma was reported by 27% physicians and intravenous pdC1-inhibitor was by 10%. The choice for long term prophylaxis included tranexamic acid, danazol and stanazolol in 29%, 22% and 8% respectively.

**Conclusions:** This epidemiological survey of HAE in India provides useful information about total number of diagnosed patients with HAE in India and the existent practices of diagnosis and management of HAE amongst physicians. The survey suggests a large number of patients with HAE in India still remain undiagnosed. The survey would be continued and ongoing efforts are being put to diagnose as many patients as possible with HAE in the country.

## O-15

### HAE with normal C1-INH (HAE-n C1-INH) and spontaneous mast cell AE (AE-MC): clinical similarities and differences

Alexis Bocquet, Delphine Gobert, Isabelle Boccon-Gibod, Olivier Fain, David Launay, Laurence Bouillet

Internal Medicine, French National Reference Centre for Angioedema, Grenoble, France

Allergy, Asthma & Clinical Immunology 2026, 22(Suppl 1):O-15

**Introduction:** HAE-nC1-INH are difficult to diagnose. Indeed, their clinical presentation can be comparable to that of isolated spontaneous AE-MC (without associated urticaria). The discovery of new pathological variants associated with HAE-nC1-INH on one hand (FXII, PLG, KNG…), and the use of omalizumab in AE-MC resistant to anti-histamines on the other hand, has enabled many diagnoses to be rectified in recent years. We propose to study the clinical similarities and differences of these AEs in the light of corrected diagnoses.

**Methods:** This is a multicenter retrospective study of patients who have consulted the CREAK network since 2005. All patients labelled HAE-nC1-INH were positive for either a pathogenic FXII or PLG variant. All AE-MC had improved by more than 90% on anti-H1 or omalizumab. AE that do not meet the diagnostic criteria for HAE-nC1-INH and AE-MC are referred to as AE-UNK.

**Results:** We included 53 patients with AE-MC, 19 with HAE-nC1-INH (FXII and PLG) and 10 with AE-UNK. Most were women (83.53%). The mean age at first attack was 28 ± years. The reason for the first consultation at CREAK was an AE in 76.5% of cases, or recurrent abdominal pain in 25.9%. An atopic context was present in 53% of AE-MC, 22.2% of HAE-n C1-INH and 33.3% of AE-UNK.

Family history of AE was more frequent in HAE-nC1-INH (68.4%) than in AE-MC (24.5%), p < 0.005. Median duration of AE was statistically different between AE-MC and HAE-nC1-INH patients: 36 h ± 25.3 versus 72 h ± 30.41 (p = 0.012). Attacks frequency was statistically higher in AE-MC (4 ± 4.89 per month) than in HAE-nC1-INH (2 per month ± 1.4) (P < 0.01). The increase in attacks frequency during pregnancy was statistically more frequent in HAE-nC1-INH women (100%) than in AE-MC (60%), p < 0.05. Abdominal pain was reported by 41.5% of AE-MC and 68.4% of HAE-nC1-INH (p = 0.059). 58.3% of AE-MC and 56.2% of HAE-nC1-INH reported AE during their first estrogen-progestin pill (p = 0.097).

100% of AE-MC patients vs 52.63% of HAE-nC1-INH and 45.4% versus 26.3% patients reported that corticosteroids and anti H1 were ineffective during an attack respectively.. 26.3% of AE-MC and 83.3% of HAE-nC1-INH felt that icatibant was effective in treating their attacks. Long-term tranexamic acid (TA) reduced attack frequency by more than 75% in 25% of AE-MC and 50% of HAE-n C1-INH (p not significant). In 37.7% of AE-MC, spontaneous urticaria appeared during follow-up, with a median delay of 3.5 years ± 8.1 after the first AE.

**Conclusion:** There are many clinical similarities between HAE-nC1-INH and AE-MC, such as the location of AE, the presence of painful abdominal attacks, the occurrence of the first attack under CO, and history of acute urticaria. Therapeutic responses (to corticoids, anti-H1, icatibant for acute attacks and TA for long term prophylaxis) are not clinically relevant to the diagnosis. It is essential to validate the diagnosis of HAE-nC1-INH by searching for pathological variants (FXII, PLG KNG…). However, family history of AE, worsening of attacks during pregnancy, and duration of attacks beyond 72 h are elements in favor of HAE-nC1-INH.

## O-16

### Complement characteristics in angioedema due to acquired C1-inhibitor deficiency: A clinico-biological correlation

Leo Guennoun^1^,*, Federica Defendi^2^, Guillaume Armengol^3^, Isabelle Boccon Gibod^4^, Laurence Bouilllet^4^, Pierre Yves Jeandel^5^, David Launay^6^, Luminita Luca^7^, Olivier Fain^1^, Delphine Gobert^1^

^1^Sorbonne Université, Service de médecine interne, AP-HP, Hôpital Saint Antoine, Paris, France; ^2^Service d’immunologie, Hôpital universitaire de Grenoble, Grenoble, France; ^3^Service de médecine interne, Centre Hospitalier Universitaire, Rouen, France; ^4^Centre national de référence des angioœdèmes (CREAK), Service de médecine interne, Hôpital universitaire de Grenoble, Grenoble, France; ^5^Service de médecine interne, Hôtel Hospitalier—Magnan, Nice, France; ^6^Service de médecine interne, Centre Hospitalier Universitaire Claude-Huriez, Lille, France; ^7^Service de médecine interne et Maladies Infectieuses, Centre Hospitalier Universitaire, Poitiers, France

*leo.guennoun@aphp.fr

Allergy, Asthma & Clinical Immunology 2026, 22(Suppl 1):O-16

**Background:** Angioedema due to acquired C1-inhibitor deficiency (AAE-C1-INH) is a rare condition, frequently associated to indolent lymphoma and monoclonal gammopathies of undetermined significance (MGUS). The diagnosis relies on recurrent angioedema after the age of 30, no familial history of angioedema, and functional C1-INH below 50% of the reference value. In France, the national registry of AAE-C1-INH includes 162 patients to date. The complement analysis can be performed in various immunology laboratories, and the published series provided data irrespective of the laboratory that performed them. Moreover, the clinical correlations to complement parameters are poorly understood.

**Material and methods:** We retrospectively studied the medical files of patients included in the national registry of AAE-C1-INH who had been diagnosed in the immunology laboratory of the national reference center of kinin angioedema (CREAK), located in Grenoble, to ensure reproducibility, and analyzed clinico-biological correlations at diagnosis and during follow-up.

**Results:** We included 104 patients with functional C1-INH below 50% of the reference value. Complement assessment was performed 0.52 (0.12–1.44) years after angioedema’s first attack. A hematological disease was present in 76 patients (72%): 54 lymphomas (52%) and 22 MGUS (21%). At diagnosis, C1-INH protein was decreased in 86% of patients. Anti-C1-INH antibodies were present in 45% of patients. Two subgroups emerged: patients with lymphomas had a lower C1-INH protein, C1q and C4, whereas patients with no hematological condition more frequently had normal C1-INH protein and anti-C1-INH antibodies. Follow up evaluation was available in 59 patients (57%), after a median duration of 3 years (0.5–10). Functional C1-INH normalization correlated with remission of angioedema, and with efficient treatment of an underlying condition. Among 5 patients treated with rituximab for anti-C1-INH antibodies associated AAE-C1-INH, without lymphoma, only one had improvement of angioedema, and none had a disappearance of antibodies.

**Conclusions:** Complement characteristics at diagnosis of AAE-C1-INH depend on the underlying hematological disease, with greater involvement of the classical pathway in patients with lymphoma. Our work identified a clinico-biological correlation: normalization of complement associates with disappearance of angioedema attacks. Treatment of lymphoma leads to clinico-biological remission, but rituximab is not effective in MGUS-associated and idiopathic AAE with anti-C1-INH Abs. The persistence of low C1-INH value in patients in remission for angioedema suggests that other mechanisms are involved in the occurrence of angioedema attacks.

Acknowledgements: The French reference centers network of bradykinic angioedema (CREAK)

## O-18

### Hereditary angioedema: A national investigation of associated comorbidities and surgical procedures

Lili Voloncs-Mindszenthy^1^, Hanga Réka Horváth^1^, Noémi Andrási^1,2,3^, Tamás Szilágyi^4^, Henriette Farkas^1^

^1^Hungarian Angioedema Center of Reference and Excellence, Department of Internal Medicine and Haematology, Semmelweis University, Budapest, Hungary; ^2^Doctoral School, Semmelweis University, Budapest, Hungary; ^3^Pediatric Center, Tűzoltó street Department, Semmelweis University, Budapest, Hungary; ^4^Department of Orthopædics and Traumatology, St. John's Hospital, Budapest, Hungary

Allergy, Asthma & Clinical Immunology 2026, 22(Suppl 1):O-18

**Background:** Hereditary angioedema due to C1-inhibitor deficiency (HAE-C1-INH) is characterized by recurrent and unpredictable subcutaneous and/or submucosal angioedema attacks. C1-inhibitor is a serine protease inhibitor that plays a regulatory role in several plasma enzyme cascade systems, implicating its crucial function in various processes. The absence of this control raises concerns about the potential development of comorbidities in HAE-C1-INH.

We aimed to investigate the full range of comorbidities present in the Hungarian HAE-C1-INH patient population and to examine the surgical procedures performed on these patients. Additionally, we sought to compare our results to those of the Hungarian general population.

**Patients and methods:** We performed a long-term prospective longitudinal study on 178 Hungarian adult HAE-C1-INH patients followed up at the Hungarian Angioedema Center of Reference and Excellence between 1979 and 2023. We used the demographic, clinical, laboratory and radiographic data recorded in our comprehensive registry for the examination of comorbidities and surgical procedures. Information about the general Hungarian population was extracted from national and European statistical databases, as well as from individual articles.

**Results:** At least one comorbidity was present in 159 of our patients. From the observed 51 diseases, headache (58%), hypercholesterolemia (52%), hypertension (30%), and liver diseases (26%) were the most prevalent. Hypercholesterolemia and depression/anxiety were at least three times more common in the HAE-C1-INH population as compared to the Hungarian general population. Tonsillectomy was performed 32, appendectomy 11, and inguinal hernioplasty 2 times more often before the diagnosis of HAE-C1-INH was established. Every surgical procedure was more prevalent in the HAE-C1-INH population.

**Conclusion:** Regular, targeted screening is indispensable for the prevention and timely diagnosis of certain conditions found in higher prevalence in the HAE-C1-INH population. The early identification and adequate treatment of angioedema attacks help prevent avoidable surgical interventions.

## O-19

### Clinical expressions, disease course, quality-of-life and resilience in subgroups of patients with angioedema

Iris Leibovich-Nassi^1,2,3^,*, Avner Reshef^2^, Michal Itzhaki^3^

^1^Academic School of Nursing, Barzilai University Medical Center, Ashkelon, Affiliated to Ben-Gurion University of the Negev, Beer-Sheva, Israel; ^2^Allergy, Immunology & Angioedema Research Center, Barzilai University Medical Center, Ashkelon, Affiliated to Ben-Gurion University of the Negev, Beer-Sheva, Israel; ^3^Department of Nursing Sciences, School of Health Professions, Faculty of Medical and Health Sciences, Tel Aviv University, Tel Aviv, Israel

*irisl@bmc.gov.il

Allergy, Asthma & Clinical Immunology 2026, 22(Suppl 1):O-19

**Background:** Angioedema (AE) manifests by recurrent tissue edema attacks, attributed to increased vascular endothelial hyper-permeability. Affected patients suffer from recurrent episodes of swelling of the face, extremities, genitals, upper airways, and abdominal pains. Attacks are unpredictable and potentially life-threatening. In addition to the two known genetic mutations leading to C1-inhibitor deficiency (HAE-C1-INH Types 1&2), recently described genetic variants in individuals and families with recurrent AE, have been found and labeled HAE-nC1-INH. Patients with AE with unknown causes or genetic mutations comprise a heterogeneous population (HAE-UNK). As a lifetime chronic disease, AE affects considerably the patient's quality of life, coping competence, and resilience. Few studies have compared the disease trajectory and personal journey of patients with different types of AE.

**Methods:** A cross-sectional prospective study consisting of three groups was conducted: A: HAE-C1-INH (Types 1&2), B: HAE-FXII (all Factor XII mutation), and C: AE-UNK. Study instruments were based on validated questionnaires (HAE-EPA) previously utilized in HAE studies, and the Connor-Davidson Resilience Scale (CD-RISC). The Local Ethics Committee approved the study, and all subjects signed an informed consent.

**Results:** The study included 111 patients, 68.5% were females. The mean age was 41.7 ± 13.6 years, mean age of onset 44.5 ± 17.4 years, and mean age of diagnosis was 19.58 ± 15.23. Groups A, B, and C consisted of 72 (64.9%), 16 (14.4%), and 23 (20.7%), respectively. Most patients (58.55%) received medications. Patients rated their coping with the illness at a moderate level of adaptation, and the level of resilience was high. Significant differences between the groups were found. Age of onset was earlier in group A (Mean 10.31 ± 10.46 years) than B (19.70 ± 3.72) and C (30.34 ± 15.20) (F = 28.31, p < 0.001). Similarly, mean age at diagnosis was earlier in A (13.85 ± 12.83 years) compared to B (24.93 ± 7.51) and C (34.73 ± 14.04) (F = 25.03, p < 0.001). Familial aggregation was significantly greater in A (5.35 ± 4.21) and B (5.83, ± 1.57) compared to C (0.60 ± 1.57) (F = 6.47, p = 0.002). Patients in group A reported the highest coping ability (7.88 ± 2.44) compared to B (M = 5.13 ± 3.07) and C (5.48 ± 2.81) (F = 41.6, p < 0.001). Mean HRQoL scores were higher in A (8.47 ± 2.06) than in C (7.74 ± 1.76) (F = 13.0, p < 0.001), and resilience was lowest in C (3.74 ± 0.67) (F = 4.75, p < 0.001).

**Conclusions:** Clinical features, disease course, HRQoL, and the patient's personal journey vary among the three designated AE groups, which calls for further investigation.

## O-20

### Triggers in focus: A deep dive into what sets off HAE attacks

Adil Bahadir^1^*, Emine Nihal Mete Gokmen^1^, Asuman Çamyar^2^, Meryem Demir^1^, Melih Özışık^1^, Gökten Bulut^1^, Ecem Ay^1^, Kutay Kırdök^1^, Ümitcan Ateş^1^, Hasibe Aytaç^1^, Züleyha Galata^1^, Kasım Okan^1^, Hatice Serpil Akten^1^, Gülhan Demiroğlu^1^, Ceyda Tunakan Dalgıç^1^, Ragıp Fatih Kural^1^, Zeynep Peker Koç^1^, Reyhan Gümüşburun^1^, Sinem İnan^1^, Sevgi Altay^1^, Emel Dilek^1^, Onurcan Yıldırım^1^, Aylin Okumuş^1^, Yusuf Özeke^1^, Aytül Zerrin Sin^1^

^1^Ege University Faculty of Medicine, Izmir, Turkey; ^2^Bakircay University Cigli Regional Education Hospital, Izmir, Turkey

*adilbahadir.md@outlook.com

Allergy, Asthma & Clinical Immunology 2026, 22(Suppl 1):O-20

**Background:** Hereditary angioedema's recurrent attacks can be spontaneous or triggered. In this study, we aim to investigate the differences in attack triggers according to age, gender and disease onset age.

**Methods:** This retrospective cross-sectional study analyzes the attack triggering factors' statistics on 155 of our hospital’s patients (93 female and 62 male), with a median age of 43 (9–90), and a median age of disease onset as 11 (1–51), median diagnosis delay of 18 years (0–58). The HAE subtypes in our study were Type 1 (82.4%) and Type 2 (17.6%). Data were collected through electronic health records. The inclusion criteria were informed consent, HAE Type 1 or Type 2 diagnosis, and not having a mental disorder. Evaluated factors are menopause, pregnancy, menstruation, oral contraceptive pill (OCP) use, vaginal birth, C-section, minor surgery, major surgery, puberty, infection, physical activity, sleep deprivation, physical trauma, emotional stress in our study. Major surgery involves organ excision or extensive tissue removal via body cavity entry, whereas minor surgery is limited to superficial tissues without structural alteration. Statistical analyses used IBM SPSS Statistics, employing Chi-square (χ2) and Fisher’s exact test for categorical variables, and the Mann–Whitney U test for non-normally distributed data. A p-value < 0.05 was deemed significant.

**Results:** The most common triggers were emotional stress (83,2%), physical trauma (82,6%), physical activity (71,1%), pregnancy (48,4%), menstruation (40,9%), infection (32,9%), and OCP use (24,7%). Major surgery (13.5%) was found a highly significant trigger between genders, with women having a higher tendency to experience attacks after a major surgery (p < 0.001), as 20.4% of women and 3.22% of men experienced. Infection was a significantly stronger trigger in patients with disease-onset at 0–10 years (44.6%) compared to 11–20 years (22%) (p < 0.05). Between HAE Type 1 and Type 2, sleep deprivation has a statistically significant difference (p < 0.05). 0.8% of Type 1 patients identified sleep deprivation as a trigger, while this rate is 4.2% in Type 2 patients.

**Conclusion:** Our study showed that emotional stress, physical trauma, and physical activity were the most common attack triggers among the HAE patient population. Major surgery was found to trigger more attacks in women, infection was a stronger trigger in those with disease onset between the ages of 0 and 10, and sleep deprivation was a more pronounced trigger factor among Type 2 patients. These findings emphasize the importance of individualized treatment methods and the value of comprehensive attack prevention strategies.

## O-21

### Angiotensin converting enzyme inhibitor angioedema in an African population, a case control cohort

Cascia Day^1,2^,*, Lovemore Mapahla^3,4^, Melissa Ribeiro^2^, Mimi Deetlefs^1^, Sian McGerty^5^, Nicola McCreesh^5^, Angelica Panieri^5^, Christian Cotchbos^5^, Robert Jeremy Freercks^5^, Adeleine Engelbrecht^6^, Sarah Pedretti^2^, Jonny Peter^1,2^**

^1^Division of Allergology and Clinical Immunology, Department of Medicine, Faculty of Health Sciences, University of Cape Town, Cape Town, South Africa; ^2^Allergy and Immunology Clinic, University of Cape Town Lung Institute, Cape Town, South Africa; ^3^The Division of Epidemiology and Biostatistics, Department of Global Health, Stellenbosch University, South Africa; ^4^The Modelling and Simulation Hub, Africa, Department of Statistical Sciences, University of Cape Town, Cape Town, South Africa; ^5^Livingstone Tertiary Hospital, Stanford Road, Korsten, 6020, Gqeberha, Eastern Cape, South Africa; ^6^District 6 Day Hospital, Western Cape, Cape Town, South Africa

*cascia.day@uct.ac.za

**jonny.peter@uct.ac.za

Allergy, Asthma & Clinical Immunology 2026, 22(Suppl 1):O-21

**Introduction:** Angiotensin converting enzyme inhibitors (ACE-I) have proven mortality and morbidity benefit in hypertension, ischemic heart disease, heart failure, and renal disease – and they are one of the most prescribed medications globally. ACE-I angioedema (ACEI-AE) is a potentially life-threatening adverse drug reaction that occurs in 0.7–1% of ACE-I users; it is the single most common cause of angioedema presenting to emergency centers, with increasing cases around the world. However, the clinical profile of ACE-I angioedema is poorly charcterised in African populations.

**Methods:** A case-controlled cohort study with prospective and retrospective enrollment of ACEI-AE cases and drug-tolerant controls. Controls were defined as a patients tolerating ACEI for a minimum of two years. Cases and controls were recruited from same demographic areas, including both hospitals and clinics. Information regarding demographics and clinical history was captured.

**Results:** A total of 249 ACE-I AE cases, and 476 ACE-I tolerant controls were enrolled, 24 cases were excluded as they had evidence of drug hypersensitivity, with 225 cases remaining. In the cohort the median age was 58 years (IQR 47;67), 57.7% were female (p = 0.059), and 38.5% (266/691) had Fitzpatrick V-VI skin tone. The cases and controls were similar in terms of age and gender, almost all had hypertension, and there were no differences with respect to previously reported risk factors such as older age, allergic rhinitis, and immunosuppression use. ACEI-AE occurred less than 30 days in only 31.1% (70/225), with median of 82.8 months (IQR 34.4; 156.4 months) on an ACE-I before developing angioedema—with one patient taking ACE-I for 40 years before developing angioedema. All the ACE-I AE cases had swelling above the shoulders, most commonly involving the lips in 73.3% (165/225), and tongue 50.7% (114/225). Most of the ACE-I angioedema cases occurred in summer (78/192, 40.6%), and spring (51/192, 26.6%). Overall, 80.4% of ACE-I angioedema cases required hospitalization, two required intubation, and there were no deaths.

**Conclusion:** Amongst this African population the majority of ACEI-AE occurred with a long drug latency, and with a seasonal effect. All cases involved the face and neck, and most commonly the lip and tongue. Further mechanistic work is required from African populations to understand this distinct epidemiology.

Declaration of source funding: Dr Cascia Day has received funding for her PhD from the Discovery Foundation, and the South African Medical Counsil Division of Research Capacity Development Bongani Mayosi National Health Scholars Programme. The work reported herein was made possible through funding by the South African Medical Research Council through its Division of Research Capacity Development under the Bongani Mayosi National Health Scholars Programme from funding received from the Public Health Enhancement Fund / South African National Department of Health. The content hereof is the sole responsibility of the authors and does not necessarily represent the official views of the SAMRC or the Discovery Foundation.

## O-22

### Psychometric study of specific questionnaires HAE-QoL and AE-QoL and the generic questionnaire EQ-5D-5L in adult patients with hereditary angioedema due to C1-inhibitor deficiency

Marina Lluncor-Salazar^1,2^*, Nieves Prior^3^, Donatella Lamacchia^4^, Adriana Hernanz^2^, Rosario Cabañas^1,2,5,6,7^, Itsaso Losantos^8^, **Teresa Caballero**^1,2,5,6^

^1^Allergy Department, Hospital Universitario La Paz, Madrid, Spain; ^2^Hospital La Paz Institute for Health Research (IdiPAZ), Madrid, Spain; ^3^Hospital Universitario Severo Ochoa, Leganés, Madrid, Spain; ^4^Personalized Medicine Asthma and Allergy Clinic, Humanitas Research Hospital, Milan, Italy; ^5^CSUR de Angioedema Hereditario, Hospital Universitario La Paz, Madrid, Spain; ^6^Center for Biomedical Research Network on Rare Diseases (CIBERER U754), Madrid, Spain; ^7^PIELenRED Consortium, Madrid, Spain; ^8^Department of Biostatistics, Hospital Universitario La Paz, Madrid, Spain

*mar_ls@hotmail.com

Allergy, Asthma & Clinical Immunology 2026, 22(Suppl 1):O-22

**Introduction:** The study of disease burden in patients with hereditary angioedema due to C1-inhibitor deficiency (HAE-C1-INH) includes HRQoL assessment. Several generic and specific questionnaires have been used. These tools must meet adequate measurement properties and need to be validated. The aim was to evaluate the psychometric properties of the patient reported outcomes HAE-QoL, AE-QoL and EQ5D5L in adult patients with HAE-C1-INH.

**Methods:** A prospective and observational study was performed in adult Spanish patients aged ≥ 18 with confirmed HAE-C1-INH. Psychometric properties of HAE-QoL, AE-QoL and EQ-5D-5L were evaluated. The minimal clinically important difference (MCID) was calculated using distribution methods: 1, half the standard deviation; 2, standard error of the measurement (SEM). Statistical analysis was performed using statistical package R (version 4.3.3). The study was approved by the local ethics committee (PI-2297).

**Results:** A total of 86 patients were included in the study, median age 42.7 (IQR 35.2–58.3); 55.8% female. The three questionnaires showed low item non-response rate. Ceiling effect was observed in 6 out of 7 domains of HAE-QoL, and there was no ceiling effect in AE-QoL and EQ5D5L. Floor effect was found in 3 out of 4 domains of AE-QoL, and all domains and index value of EQ5D5L, whereas no floor effect was observed in HAE-QoL. Internal consistency (Cronbach´s alpha) was good to excellent for domains and total score of HAE-QoL (0.68–0.91 and 0.92) and AE-QoL (0.83–0.94 and 0.81), respectively, and acceptable for the index value of EQ5D5L (0.72). Test–retest fiability showed high intraclass coefficient for domains and total score of HAE-QoL (0.82–0.90, 0.86, respectively), AE-QoL (0.67–0.81, 0.79, respectively) and EQ5D5L (0.42–0.84, 0.61), except in “Self-care” domain. Convergent validity between total scores of HAE-QoL and AE-QoL was strong (0.86), whereas between total scores of HAE-QoL, AE-QoL and EQ-5D-5L index value was moderate (0.45). Construct validity was good for HAE-QoL and AE-QoL: 5 out of 6 predefined hypotheses based on clinical criteria were confirmed, whereas only 2 out of the 6 predefined hypotheses were confirmed in EQ5D5L. Discriminant validity assessment showed significant differences between severity groups for HAE-QoL, AE-QoL and EQ5D5L. The MCID of the total score of HAE-QoL was 11.5 using method 1, and 8.6, using method 2, whereas it was 11.4 and 7.0, respectively for the total score of AE-QoL and 0,1 for index value of EQ-5D-5L using both methods.

**Conclusions:** HAE-QoL, AE-QoL y EQ5D5L questionnaires have shown good psychometric properties. Construct validity was limited for EQ5D5L.

## O-23

### Long-term efficacy and safety of prophylactic treatment with once-daily deucrictibant for angioedema due to acquired C1-inhibitor deficiency: Final study results

Mats de Lange MD^1^, Remy S. Petersen MD PhD^1^, Lauré M. Fijen MD PhD^1^, Danny M. Cohn MD PhD^1^

Amsterdam UMC, Department of Vascular Medicine, Amsterdam Cardiovascular Sciences, University of Amsterdam, Amsterdam, The Netherlands

Allergy, Asthma & Clinical Immunology 2026, 22(Suppl 1):O-23

**Background:** Acquired angioedema due to C1-inhibitor deficiency (AAE-C1-INH) is a rare disease with an estimated prevalence of 1 in 500,000. Insufficient inhibition of the kallikrein-kinin system causes excessive production of bradykinin, leading to episodes of angioedema in subcutaneous and submucosal tissues. Unlike hereditary angioedema, AAE-C1-INH typically manifests later in life and is often associated with underlying conditions, such as plasma cell dyscrasias, lymphoproliferative disorders or autoimmune diseases. Current long-term prophylactic treatment strategies for AAE-C1-INH are primarily based on descriptive, case-based evidence. A previous randomized, placebo-controlled trial (EudraCT 2021–000720-36) demonstrated that daily use of the oral bradykinin B2 receptor antagonist deucrictibant immediate-release capsule effectively prevented angioedema attacks in patients with AAE-C1-INH. An extended-release formulation of once-daily deucrictibant tablet is currently under development for long-term prophylactic use in hereditary angioedema and in AAE-C1-INH.

**Objective:** To investigate the long-term efficacy and safety of deucrictibant extended-release tablet as long-term prophylaxis in patients with AAE-C1-INH.

**Methods:** The ONCE-AID trial, an open-label, single-arm trial with once-daily deucrictibant 40 mg extended-release tablets was conducted.

**Results:** Four patients with AAE-C1-INH were enrolled, with an on-treatment follow-up duration in this study ranging from 563 to 612 days. Three patients rolled over from the previous trial with deucrictibant. The mean monthly (per four weeks) attack rate at baseline was 1.2, 1.2, 0.9, and 2.1, respectively (mean 1.35). The baseline attack rate covers 90 days prior to randomization for prophylactic treatment in the randomized controlled trial for the roll-over patients, and 90 days prior to enrolment in the open-label study for the fourth patient. During daily treatment with deucrictibant the mean monthly attack rate for all patients combined was 0.01. One mild abdominal attack was reported by one patient, two days after initiation of deucrictibant treatment. The other three patients were attack-free during treatment with deucrictibant. All four patients showed clinically meaningful improvements in the ‘Angioedema Control Test’ and ‘Angioedema Quality of Life Questionnaire’ scores. Median ‘Treatment Satisfaction Questionnaire for Medication version II’ scores were above 90 throughout the study for each domain, indicating high treatment satisfaction. No drug-related adverse events were reported. During treatment, one serious adverse event occurred, involving hospital admission for diagnostic evaluation of a suspected lymphoma. This event resolved without lasting complications and was deemed unrelated to the study medication.

**Conclusion:** Deucrictibant extended-release tablet effectively prevented angioedema attacks in patients with acquired C1-inhibitor deficiency, without safety concerns.

## O-24

### Garadacimab-mediated inhibition of activated factor XII had no impact on haemostasis in patients with hereditary angioedema

Timothy J. Craig^1,2^, Danny M. Cohn^3^, Markus Magerl^4,5,6^, H. Henry Li^7^, Harsha Shetty^8^, Maressa Pollen^8^, John-Philip Lawo^9^, Emel Aygören-Pürsün^10^

^1^Allergy, Asthma and Immunology, Departments of Medicine, Pediatrics, OB/Gyn/MFM and Biomedical Sciences, Pennsylvania State University, Hershey, PA, USA; ^2^Vinmec International Hospital, Hanoi, Vietnam; ^3^Department of Vascular Medicine, Amsterdam Cardiovascular Sciences, Amsterdam UMC, University of Amsterdam, Amsterdam, The Netherlands; ^4^Institute of Allergology, Charité–Universitätsmedizin Berlin, Berlin, Germany; ^5^Freie Universität Berlin and Humboldt-Universität zu Berlin, Berlin, Germany; ^6^Fraunhofer Institute for Translational Medicine and Pharmacology (ITMP), Immunology and Allergology, Berlin, Germany; ^7^Institute for Asthma and Allergy, Chevy Chase, MD, USA; ^8^CSL Behring, King of Prussia, PA, USA; ^9^CSL Innovation GmbH, Marburg, Germany; ^10^University Hospital Frankfurt, Department for Children and Adolescents, Goethe University Frankfurt, Frankfurt, Germany

Allergy, Asthma & Clinical Immunology 2026, 22(Suppl 1):O-24

**Background:** Garadacimab (anti-activated factor XII [FXIIa] antibody) for hereditary angioedema (HAE) long-term prophylaxis (LTP) has demonstrated early and durable efficacy with a favourable long-term safety profile. The impact of garadacimab-mediated inhibition of FXIIa (principal initiator of the kallikrein–kinin system and intrinsic coagulation pathway) on haemostasis was evaluated in the clinical programme, which included patients at potential haemostatic risk due to undergoing concomitant surgical/medical procedures, or co-administration of haemostasis-impacting treatments (anticoagulants, antiplatelets or factor Xa inhibitors).

**Materials and methods:** Three analyses were conducted. Firstly, data from HAE studies (Phase 2, pivotal Phase 3 and ongoing Phase 3 open-label extension [OLE; NCT04739059; data cutoff: February 13, 2023]) were analysed using Standardised MedDRA Query (SMQ); events screened by SMQ (including investigator-reported events) were medically reviewed for abnormal bleeding. Secondly, data from patients who underwent surgical/medical procedures in the Phase 3 OLE (data cutoff: June 16, 2023) were assessed for abnormal bleeding events (included within the adverse events of special interest per protocol definition, as assessed by the investigator). Thirdly, abnormal bleeding was assessed in patients receiving garadacimab co-administered with haemostasis-impacting treatments in the three HAE studies (Phase 3 OLE data cutoff: June 16, 2023) and Phase 2 COVID-19 study (NCT04409509).

**Results:** Medical review of SMQ-screened events revealed no abnormal bleeding with garadacimab LTP (n = 172) or placebo (n = 33). In the Phase 3 OLE (N = 161), 25 patients underwent ≥ 1 surgical/medical procedure (35 total); none experienced abnormal bleeding during treatment. Procedures included dental treatments (16/35, 46%), ophthalmologic surgeries (5/35, 14%), endoscopic investigations (3/35, 9%), muscle-repair (2/35, 6%) and toe (2/35, 6%) surgeries, partial lung resection (1/35, 3%), portacath removal (1/35, 3%) and other procedures (cyst surgery; biopsy; naevus, basal cell carcinoma or syringoma removal: 5/35, one procedure each, 14%). In the HAE studies, no abnormal bleeding was observed in patients who received garadacimab LTP co-administered with haemostasis-impacting treatments (one-off or long term). In the COVID-19 study, three bleeding events (epistaxis, n = 3/58 patients, none garadacimab related) were reported with garadacimab and four bleeding events (haemorrhagic stroke, subarachnoid haemorrhage, thrombocytopenia and subdural haematoma, n = 1/59 patients) with placebo. No garadacimab-related abnormal bleeding was observed despite 95% of patients (n = 55/58) being co-administered haemostasis-impacting treatments.

**Conclusions:** Garadacimab-mediated FXIIa inhibition had no impact on haemostasis in patients co-administered with haemostasis-impacting treatments or undergoing surgical/medical procedures. No garadacimab-related abnormal bleeding was observed in the COVID-19 study. Garadacimab demonstrated a well-tolerated safety profile in patients with HAE and COVID-19 bearing potential haemostatic risk.

## O-25

### Lanadelumab shows infrequent injection site reactions and high patient satisfaction in patients with HAE: ENABLE Study Results

Mauro Cancian^1^, Walter Wuillemin^2^, Inmaculada Martinez-Saguer^3^, Andrea Zanichelli^4,5^, Robin Lochbaum^6^, Susanne Trainotti^7^, Daniel Nova Estepan^8^, Irmgard Andresen^9^,*, Maureen Watt^8^, Natalie Khutoryansky^8^, Tamar Kinaciyan^10^

^1^Department of Systems Medicine, University Hospital of Padua, Padua, Italy; ^2^Division of Hematology and Hematology Laboratory, Department of Internal Medicine, Luzerner Kantonsspital, Lucerne and University of Berne, Berne, Switzerland; ^3^HZRM Hemophilia Center Rhine Main, Frankfurt, Germany; ^4^Operative Unit of Medicine, Angioedema Center, IRCCS Policlinico San Donato, San Donato, Milanese, Milan, Italy; ^5^Dipartimento di Scienze Biomediche per la Salute, University of Milan, Milan, Italy; ^6^Department of Otorhinolaryngology, Head and Neck Surgery, Ulm University Medical Center, Ulm, Germany; ^7^Department of Otorhinolaryngology, TUM University Hospital, Klinikum rechts der Isar, Technical University of Munich, Munich, Germany; ^8^Takeda Development Center Americas, Inc., Lexington, MA, USA; ^9^Takeda Pharmaceuticals International AG, Zurich, Switzerland; ^10^Department of Dermatology, Medical University of Vienna, Vienna, Austria

*irmgard.andresen@takeda.com

Allergy, Asthma & Clinical Immunology 2026, 22(Suppl 1):O-25

**Background:** We report the safety and patient satisfaction with Lanadelumab treatment in patients with hereditary angioedema (HAE) from the final analysis of the Phase IV, non-interventional, prospective ENABLE Study evaluating the long-term real-world effectiveness of Lanadelumab.

**Methods:** Patients aged ≥ 12 years with HAE who initiated Lanadelumab treatment according to approved product labelling were recruited from Austria, Germany, Israel, Italy, Kuwait, Spain, and Switzerland and followed for up to 36 months. Safety was evaluated by assessing treatment-emergent adverse events (TEAEs) captured by electronic case report forms. Treatment satisfaction in adult (≥ 18 years) patients was assessed by responses to the Treatment Satisfaction Questionnaire for Medication-9 (TSQM-9) collected using a smartphone application at baseline (defined as the last observed value before Lanadelumab initiation), Months 1, 2, 3, 6, 12, and 12-monthly thereafter until the end of follow-up.

**Results:** In total, 138 (mean ± SD age 41.0 ± 14.4 years, 5.8% aged < 18 years, 62.3% female, 92.0% HAE-C1-INH-Type1) of the 139 enrolled patients were included for analysis. Patients received Lanadelumab 300 mg every 2 weeks at enrolment and many reduced the dosing frequency over the follow-up period; median (range) treatment duration was 744.5 (57–1133) days. Ninety-five of 138 (68.8%) patients experienced ≥ 1 TEAE during the study period, for a total of 474 TEAEs, no TEAEs were fatal. Twenty-five of 138 (18.1%) patients reported 84 treatment-related TEAEs (TR-TEAEs), of which 63 events were injection site reactions (ISRs). Seven of 138 patients (5.1%) reported injection site erythema (18 events) and 6/138 (4.3%) reported injection site pain (23 events). No Lanadelumab-related ISRs reported were severe or serious, most (51/63; 81.0%) were mild. Of patients with TEAEs in the first 120 days following Lanadelumab initiation, 9/25, 8/30, 2/11, and 13/88 had TR-TEAEs during Days 0–30, Days 31–90, Days 91–120, and after Day 120, respectively. Most TR-TEAEs were of mild-to-moderate severity and non-serious. No TR-TEAEs led to Lanadelumab discontinuation. Among adult patients who completed the TSQM-9, mean ± SD Global Satisfaction, Effectiveness, and Convenience Scores increased from 68.2 ± 21.5, 64.7 ± 21.9, and 65.1 ± 21.6 at baseline (n = 120) to 77.7 ± 22.6, 77.8 ± 26.6, and 79.5 ± 19.0 at Month 1 (n = 95), 86.6 ± 17.7, 79.0 ± 32.2, and 84.6 ± 16.5 at Month 12 (n = 97), and 93.7 ± 13.7, 79.8 ± 36.2, and 86.8 ± 19.8 at Month 36 (n = 35), respectively.

**Conclusions:** Findings from this real-world study of patients with HAE receiving Lanadelumab show a consistent safety profile with previous studies, no new safety signals, and infrequent ISRs. Patient-reported satisfaction with Lanadelumab was high and increased further throughout the study period.

*Trial registration: Clinicaltrials.gov, NCT04130191.*

*Funding and acknowledgements: The ENABLE Study was sponsored by Takeda Development Center Americas, Inc., Lexington, MA, USA. Under the direction of the authors, Emily Sun, PhD, employee of Excel Scientific Solutions, Inc., provided medical writing assistance for this abstract, funded by Takeda Development Center Americas, Inc. The interpretation of the data was made by the authors independently.*

## O-26

### Reduction in percentage of days with angioedema symptoms among young children initiating berotralstat: Interim results from APeX-P

Jolanta Bernatoniene^1^, Melisande Bourgoin-Heck^2,3^, Mauro Cancian^4^, William H. Yang^5^, David Hagin^6^, Anne Pagnier^7^, Marcin Stobiecki^8^, Stéphane Gayet^9^, Tamar Kinaciyan^10^, Elsa Phillips-Angles^11^, Noémi-Anna Bara^12^, Michael DeSpirito^13^, Douglas T. Johnston^13^, Daniel Long^13^, Lindsey Noble^13^, Emel Aygören-Pürsün^14^

^1^Paediatric Immunology & Infectious Disease Department, Bristol Royal Hospital for Children, Bristol, UK; ^2^Service d'Allergologie Pédiatrique, Hôpital d'Enfants Armand-Trousseau, AP-HP Sorbonne-University, Paris, France; ^3^CREAK Hôpital Saint Antoine, AP-HP Sorbonne-University, Paris, France; ^4^Department of Systems Medicine, University Hospital of Padua, Padua, Italy; ^5^Ottawa Allergy Research Corporation and Department of Medicine, University of Ottawa, Ottawa, Ontario, Canada; ^6^Allergy and Clinical Immunology, Tel-Aviv Sourasky Medical Center and Tel-Aviv University, Tel-Aviv, Israel; ^7^Department of Pediatrics, Centre Hospitalier Universitaire de Grenoble, Grenoble, France; ^8^Department of Clinical and Environmental Allergology, Jagiellonian University Medical College, Kraków, Poland; ^9^Internal Medicine Department, La Timone University Hospital, Assistance Publique-Hôpitaux de Marseille, Marseille, France; ^10^Department of Dermatology, Medical University of Vienna, Vienna, Austria; ^11^Department of Allergy, Hospital Universitario La Paz, IdiPaz, Madrid, Spain; ^12^Centrul Clinic Mediquest/Centrul de Expertiza de Angioedem Ereditar, Sangeorgiu de Mures, Romania; ^13^BioCryst Pharmaceuticals, Inc., Durham, NC, USA; ^14^Internal Medicine and Hemostaseology, Department for Children and Adolescents, University Hospital Frankfurt, Frankfurt, Germany

Allergy, Asthma & Clinical Immunology 2026, 22(Suppl 1):O-26

**Introduction:** Symptoms of hereditary angioedema (HAE) often occur during childhood, resulting in negative impacts on daily life such as missed school days. Normalising life is one of the goals of HAE treatment, which can only be achieved with long-term prophylaxis (LTP) according to the World Allergy Organization/European Academy of Allergy and Clinical Immunology guidelines. Approved treatments for children aged < 12 years require parenteral administration; thus, an unmet need exists for targeted treatment that can be more easily administered. Berotralstat is an oral, small-molecule plasma kallikrein inhibitor for prophylaxis of HAE attacks in patients aged ≥ 12 years. Interim results from the ongoing APeX-P clinical trial, the largest study of paediatric patients with HAE, are presented.

**Methods:** APeX-P is an ongoing, open-label study evaluating the pharmacokinetics, safety, and effectiveness of berotralstat in children aged 2 to < 12 years with HAE. Most participants in APeX-P received an oral-granule formulation of berotralstat. Prior to berotralstat initiation, study participants received standard of care (SOC) treatment for 12 weeks that could include LTP, on-demand treatment, or both. A planned interim analysis was performed, including days with symptoms of angioedema and incidence of adverse events (AEs).

**Results:** Participants (N = 29) had a median age of 8.0 (range, 3–11) years, and 48.3% were female. Their median age at HAE symptom onset was 2.0 (range, 0.3–8.0) years, and most (82.8%) had symptom onset before 6 years of age. Participants reported a mean (SD) of 18.0 (20.5) days of missed school due to HAE symptoms in the previous year. During the 12-week SOC period, participants reported a mean (SD) of 11% (9.4) of days with angioedema symptoms. After 12 weeks of berotralstat treatment, they reported a mean (SD) of 4% (4.7) of days with angioedema symptoms. The most commonly reported treatment-emergent AEs were nasopharyngitis (27.6%), upper respiratory tract infection (24.1%), and headache (13.8%). No drug-related grade 3/4 AEs, drug-related serious AEs, deaths, or discontinuations related to AEs were reported.

**Conclusion:** Most participants in APeX-P missed school days in the previous year due to their disease, thereby representing a burden on patients and their parents/caregivers to live normal lives. In APeX-P, oral berotralstat was generally well tolerated and reduced the proportion of days with angioedema symptoms in a paediatric population aged 2 to < 12 years. These findings suggest that berotralstat may help normalise life in paediatric patients with HAE.

*Funding:* *This study was funded by BioCryst Pharmaceuticals, Inc.*

## O-27

### Lanadelumab’s enduring impact on HAE attack reduction and improved disease control: Final results from the ENABLE Study

Andrea Zanichelli^1,2^, Mauro Cancian^3^, Aharon Kessel^4^, Emel Aygören-Pürsün^5^, Robin Lochbaum^6^, Susanne Trainotti^7^, Daniel Nova Estepan^8^, Irmgard Andresen^9*^, Maureen Watt^8^, Natalie Khutoryansky^8^, Felix Johnson^10^

^1^Operative Unit of Medicine, Angioedema Center, IRCCS Policlinico San Donato, San Donato, Milanese, Milan, Italy; ^2^Dipartimento di Scienze Biomediche per la Salute, University of Milan, Milan, Italy; ^3^Department of Systems Medicine, University Hospital of Padua, Padua, Italy; ^4^Division of Allergy and Clinical Immunology, Bnai Zion Medical Centre, Technion Faculty of Medicine, Haifa, Israel; ^5^Department for Children and Adolescents, Angioedema Centre, University Hospital Frankfurt, Goethe University, Frankfurt, Germany; ^6^Department of Otorhinolaryngology, Head and Neck Surgery, Ulm University Medical Center, Ulm, Germany; ^7^Department of Otorhinolaryngology, TUM University Hospital, Klinikum rechts der Isar, Technical University of Munich, Munich, Germany; ^8^Takeda Development Center Americas, Inc., Lexington, MA, USA; ^9^Takeda Pharmaceuticals International AG, Zurich, Switzerland; ^10^University Hospital for Otorhinolaryngology, Head and Neck Surgery, Medical University of Innsbruck, Innsbruck, Austria

*irmgard.andresen@takeda.com

Allergy, Asthma & Clinical Immunology 2026, 22(Suppl 1):O-27

**Introduction:** ENABLE (NCT04130191), a Phase IV, non-interventional, prospective study evaluated Lanadelumab effectiveness for prevention of hereditary angioedema (HAE) attacks in real-world clinical practice. Here, we describe results from the primary objective, Lanadelumab effectiveness on patient-reported outcomes.

**Methods:** Patients with HAE aged ≥ 12 years were recruited from 18 sites across Austria, Germany, Israel, Italy, Kuwait, Spain and Switzerland. Patients who initiated Lanadelumab per product labelling were enrolled. Patient-reported HAE attacks were captured via a smartphone application-based patient- or caregiver-recorded diary. Patient perception of disease control was assessed by the Angioedema Control Test (AECT), collected by the same smartphone application at baseline (defined as the last observed value before Lanadelumab initiation), Months 1, 2, 3, 6, 12 and every 12 months thereafter until the end of follow-up (up to 36 or 24 months if enrolled before or on/after 1 March 2021, respectively).

**Results:** In total, 139 patients with HAE were enrolled, 138 of whom received ≥ 1 dose of Lanadelumab and were included in the analysis (mean age ± SD, 41.0 ± 14.4 years; 62.3% female). There were 127 patients with HAE-C1-INH-Type1. Median (range) Lanadelumab treatment duration was 26.6 (2.0–40.5) months. Mean ± SD monthly attack rates decreased by 83.9% on Lanadelumab, from 3.88 ± 3.43 attacks/month pre-Lanadelumab to 0.30 ± 0.53 on treatment. Median monthly attack rates reduced by 96.7%, from 3.0 attacks/month pre-Lanadelumab to 0.1 on treatment. Most attacks (85.9%) on Lanadelumab were mild/moderate in severity. For attacks on Lanadelumab with known locations, most affected the abdomen (572 attacks in 72/92 patients) or periphery (421 attacks in 64/92 patients). Seventy-six of 92 (82.6%) patients reported using on-demand treatment for attacks during the study. On average, patients reported poorly controlled disease (AECT score < 10) at baseline (mean ± SD, 7.5 ± 3.7). A mean AECT score ≥ 10 (indicating well-controlled disease) was achieved by Month 1 (12.5 ± 3.6) and steadily improved, reaching 14.4 ± 2.9 by Month 36 among patients with available follow-up data. The mean change in AECT score after 1 month on Lanadelumab exceeded the minimal clinically important difference of 3 points. The percentage of patients reporting well-controlled disease (AECT score ≥ 10) increased from 51.9% (67/129) at baseline to 88.3% (91/103) and 89.7% (35/39) at Months 12 and 36, respectively.

**Conclusions:** In this real-world study, patients experienced a marked and sustained reduction in HAE attack rates and meaningful increases in AECT scores from baseline by the first follow-up, which was maintained through 36 months of treatment. This reinforces the evidence for Lanadelumab as a first-line prophylaxis option.

*Trial registration: Clinicaltrials.gov, NCT04130191*

*Funding and acknowledgments: The ENABLE Study was sponsored by Takeda Development Center Americas, Inc., Lexington, MA, USA. Under the direction of the authors, Emily Sun, PhD, an employee of Excel Scientific Solutions, Inc., provided medical writing assistance for this abstract, funded by Takeda Development Center Americas, Inc. The interpretation of the data was made by the authors independently.*

## O-28

### Normalization of elevated D-dimer with donidalorsen treatment across phase 2 and phase 3 studies in patients with hereditary angioedema

Andrea Zanichelli^1,2^, Gaia Luppino^3^, Laura Bordone^4^, Sabrina Treadwell^4^, Kenneth B. Newman^4^, Sophie Wang^4^, Tao Lin^4^, Yiwen Deng^4^, Danny M. Cohn^5^

^1^Department of Biomedical Sciences for Health, University of Milan, Milan, Italy; ^2^Operative Unit of Medicina, Angioedema Center, I.R.C.C.S. Policlinico San Donato, San Donato Milanese, Milano; ^3^Otsuka Pharmaceutical Europe Ltd., Windsor, UK; ^4^Ionis, Carlsbad, CA, USA; ^5^Department of Vascular Medicine, Amsterdam Cardiovascular Sciences, Amsterdam UMC, University of Amsterdam, Amsterdam, The Netherlands

Allergy, Asthma & Clinical Immunology 2026, 22(Suppl 1):O-28

Donidalorsen, an antisense oligonucleotide targeting prekallikrein mRNA, was evaluated in a phase 2 study (CS2; NCT04030598), its open-label extension study (OLE; CS3; NCT04307381), a phase 3 study (CS5; NCT05139810), and its OLE (CS7; NCT05392114) in patients with hereditary angioedema (HAE)-C1-INH-Type1 or HAE-C1-INH-Type2. This analysis evaluated prekallikrein and D-dimer concentrations (biomarkers for hereditary angioedema attacks).

Patients received donidalorsen 80 mg/placebo every 4 weeks (Q4W) in CS2. In CS3, both CS2 placebo (index-placebo) and Q4W (index-Q4W) groups received donidalorsen Q4W. At Week 16, investigators could switch patients to donidalorsen every 8 weeks (Q8W). In CS5, patients received donidalorsen 80 mg/placebo Q4W or Q8W. In CS7, patients receiving donidalorsen/placebo Q8W in CS5 could receive donidalorsen Q4W, or Q8W if attack-free for ≥ 8 weeks. All others received donidalorsen Q4W. CS7 data are grouped by original CS5 treatment (index-Q4W/Q8W/placebo). Pharmacodynamic assessments included mean trough prekallikrein and mean D-dimer concentrations. Normal D-dimer concentration is < 0.5 μg/mL.

Twenty patients received donidalorsen / placebo in CS2; 17 patients continued into CS3. In CS5, 90 patients received donidalorsen/placebo; 83 patients continued to CS7. Mean donidalorsen exposure in CS7 was 228 days (Q4W) and 238 days (Q8W). Prekallikrein decreased by 61% (Q4W; baseline 95 mg/L) vs 3% (placebo; baseline 101 mg/L) at CS2 Week 16. At CS3 Week 156, prekallikrein decreased by 53% (index-donidalorsen) and 48% (index-placebo) compared to CS2 baseline. In CS5, prekallikrein decreased by 73% (Q4W; baseline 128 mg/L) and 47% (Q8W; baseline 144 mg/L) vs a 2% increase (placebo; baseline 118 mg/L) at Week 24. At CS7 Week 24, prekallikrein decreased by 72% (index-Q4W-to-Q4W), 36% (index-Q8W-to-Q8W), and 79% (index-placebo-to-Q4W) compared to CS5 baseline. D-dimer baseline values were 2 μg/mL (Q4W and placebo) in CS2 and decreased by 41% (Q4W) vs 18% (placebo) at Week 16. In CS3, D-dimer decreased from 3 µg/mL at baseline by 77% (index-donidalorsen) and from 4 µg/mL at baseline by 76% (index-placebo) at Week 156. In CS5, D-dimer decreased by 64% with Q4W (baseline 3 µg/mL), and increased by 3% with Q8W (baseline 0.4 µg/mL) and by 15% with placebo (baseline 2 µg/mL) at Week 24. In CS7, D-dimer decreased by 73% (index-Q4W-to-Q4W; baseline 3 µg/mL), 64% (index-Q8W-to-Q8W; baseline, 0.5 µg/mL), and 85% (index-placebo-to-Q4W; baseline, 1.2 µg/mL) at Week 24, and by 88% (index-Q4W-to-Q4W), 100% (index-Q8W-to-Q8W), and 87% (index-placebo-to-Q4W) at Week 52.

Donidalorsen reduced prekallikrein and normalised elevated baseline D-dimer concentrations in phase 2 and phase 3 studies and in long-term OLE studies.

## O-29

### Effectiveness of sebetralstat for the on-demand treatment of mucosal hereditary angio-edema attacks: Interim analysis from KONFIDENT-S

Jonathan A. Bernstein^1^, Emel Aygören-Pürsün^2^, Vesna Grivcheva-Panovska^3^, Inmaculada Martinez-Saguer^4^, Danny M. Cohn^5^, William R. Lumry^6^, Marc A. Riedl^7^, Andrea Zanichelli^8,9^, Laurence Bouillet^10^, Ya-Hsiu Chuang^11^, Michael D. Smith^11^, Christopher M. Yea^11^, Paul K. Audhya^11^, Henriette Farkas^12^*

^1^University of Cincinnati College of Medicine; and Bernstein Clinical Research Center, Cincinnati, OH, USA; ^2^University Hospital Frankfurt, Goethe University Frankfurt, Frankfurt, Germany; ^3^University Clinic of Dermatology, School of Medicine, University Saints Cyril and Methodus, Skopje, North Macedonia; ^4^HZRM Haemophilia Center Rhein Main, Frankfurt, Germany; ^5^Amsterdam University Medical Center, University of Amsterdam, Amsterdam, The Netherlands; ^6^AARA Research Center, Dallas, TX, USA; ^7^University of California, San Diego, La Jolla, CA, USA; ^8^Operative Unit of Medicine, Angioedema Center, IRCCS Policlinico San Donato, San Donato Milanese, Milan, Italy; ^9^Department of Biomedical Science for Health, University of Milan, Milan, Italy; ^10^French National Reference Center for Angioedema (CREAK), Grenoble Alpes University, Grenoble, France; ^11^KalVista Pharmaceuticals, Salisbury, United Kingdom, and Cambridge, MA, USA; ^12^Hungarian Angioedema Center of Reference and Excellence, Semmelweis University, Budapest, Hungary

*farkas.henriette@semmelweis.hu

Allergy, Asthma & Clinical Immunology 2026, 22(Suppl 1):O-29

**Background:** Attacks involving mucous membranes, including the gastrointestinal tract and the larynx, share an underlying pathophysiology. Mucosal attacks may progress rapidly and are associated with substantial morbidity. Here we present interim data on the safety and effectiveness of sebetralstat, an investigational oral on-demand therapy, in mucosal (abdominal and laryngeal) HAE-C1-INH attacks from the ongoing 2-year open-label KONFIDENT-S study (NCT05505916).

**Materials and Methods:** Participants were instructed to self-administer sebetralstat 600 mg as early as possible for each attack, regardless of severity or anatomic location. Efficacy endpoints were time to beginning of symptom relief (Patient Global Impression of Change: at least “A Little Better” at ≥ 2 consecutive time points) within 12 h; time to reduction in attack severity (Patient Global Impression of Severity [PGI-S]: improved rating at ≥ 2 consecutive time points) within 12 h; time to complete attack resolution (PGI-S rating: “None”) within 24 h. Conventional treatment administration was censored to the end of analysis window.

**Results:** As of September 14, 2024, 533 abdominal attacks (29.6% mild; 42.6% moderate; 27.8% severe/very severe) and 32 attacks involving the larynx (25.0% mild; 46.9% moderate; 28.1% severe/very severe) were treated with 1–2 administrations of sebetralstat. Treatment-related adverse events occurred in 6 (5.9%) participants experiencing an abdominal attack and 1 (6.3%) experiencing an attack involving the larynx; no difficulty swallowing sebetralstat was reported. Median (IQR) time to treatment was 20.0 min (1.0–61.0) and 11.5 min (1.0–34.0) for abdominal and laryngeal attacks, respectively. Median times to beginning of symptom relief were 1.3 h (0.76–3.54) for abdominal attacks and 1.3 h (0.76–3.02) for laryngeal attacks; reduction in attack severity, 3.5 h (1.26- > 12) and 4.3 h (1.22- > 12); complete attack resolution, 15.2 h (4.46- > 24) and 12.7 h (5.11- > 24). Within 12 h, 95 (17.8%) abdominal attacks and 4 (12.5%) attacks involving the larynx were treated with an additional sebetralstat administration. Within 12 h, 43 (8.1%) abdominal attacks and 3 (9.4%) attacks involving the larynx were treated with conventional treatment. Of attacks reaching beginning of symptom relief within 12 h (85.7% of abdominal; 89.3% of attacks involving the larynx), 95.8% and 96.0%, respectively, did so before or without an additional sebetralstat administration.

**Conclusions:** In this interim analysis of the ongoing KONFIDENT-S study, sebetralstat enabled rapid treatment, was well-tolerated, and demonstrated similar effectiveness for both forms of mucosal attacks, abdominal and laryngeal.

## O-30

### Navenibart for hereditary angioedema (HAE): Analysis of safety, pharmacokinetic, and pharmacodynamic data from Phase 1a and Phase 1b/2 ALPHA-STAR trial

Marc Riedl^1^,*, Raffi Tachdijan^2^, Michele Gunsior^3^, Ganesh Mugundu^3^, Theodora Cohen^3^, Christopher Morabito^3^, William Lumry^4^

^1^University of California, San Diego, La Jolla, CA, USA; ^2^University of California, Los Angeles, Santa Monica, CA, USA; ^3^Astria Therapeutics, Inc., Boston, MA, USA; ^4^Medical City Children's Hospital, Dallas, TX, USA

*mriedl@health.ucsd.edu

Allergy, Asthma & Clinical Immunology 2026, 22(Suppl 1):O-30

**Background:** Hereditary angioedema (HAE) is a rare, autosomal dominant disease characterised by unpredictable attacks that impact quality of life. Navenibart (STAR-0215) is an investigational, long-acting monoclonal antibody plasma kallikrein inhibitor. Navenibart has been studied in Phase 1a (NCT05477160) in healthy subjects and Phase 1b/2, ALPHA-STAR (NCT05695248) in patients with HAE. A global Phase 3 trial, ALPHA-ORBIT (NCT06842823) is currently ongoing.

**Methods:** Safety, pharmacokinetics (PK), and pharmacodynamics (PD) were evaluated in adults in both trials. Phase 1a (n = 41; 31 navenibart / 10 placebo) investigated single ascending doses of navenibart in healthy subjects, including subcutaneous (SC) doses ranging from 100 to 1200 mg and a 600 mg intravenous dose. Phase 1b/2 (target enrolment: n = 16) assessed multiple SC dosing cohorts: 1) 450 mg, 2) 600 mg / 300 mg on Day 84 and 3) 600 mg / 600 mg on Day 28 in participants with HAE.

The primary endpoint in both trials was the incidence of treatment emergent adverse events (TEAEs). Concentrations of navenibart were measured by validated immunoassays. PD assessments measured plasma kallikrein activity by changes in cleaved high molecular weight kininogen (cHWMK) via western blot. Here, we describe safety, PK, and PD results.

**Results:** In Phase 1a, TEAEs occurred in 21 (68%) navenibart and 6 (60%) placebo subjects. In Phase 1b/2, TEAEs occurred in 15 (94%) participants. Navenibart-related TEAEs occurred in 11 Phase 1a and 3 Phase 1b/2 participants, most commonly injection site reactions (all mild), with no dose or concentration dependence. There were no reports of pain, or clinically relevant changes in laboratory (including aPTT) or ECG parameters. No serious TEAEs and no treatment discontinuations were reported. Navenibart peak concentrations were achieved in 8–11 days in all cohorts. In the Phase1b/2 trial, mean cHMWK decreased by ≥ 50% in 7–14 days and was durable.

**Conclusion:** Navenibart was well-tolerated in the Phase 1a and Phase 1b/2 trials with no dose- or concentration-related TEAEs. Navenibart achieved maximum concentra-tions rapidly after a single SC dose in all cohorts with slow elimination. Navenibart rapidly and durably reduced plasma kallikrein activity after single and multiple doses for at least 6 months in patients with HAE. These results support the doses selected for the ongoing Phase 3 trial (600 mg, then 300 mg Q3 months; 600 mg Q6 months; and 600 mg Q3 months).

## O-31

### HAELO, a phase 3, global, randomised, double-blind, placebo-controlled study of NTLA-2002, a CRISPR-based gene editing therapy, in participants with hereditary angioedema

Emel Aygӧren-Pürsün^1^, Aleena Banerji^2^, Danny M. Cohn^3^, Timothy J. Craig^4^, Henriette Farkas^5^, Hilary J. Longhurst^6^, William R. Lumry^7^, Markus Magerl^8^, Marc A. Riedl^9^, Adele Golden^10^, Mrinal Y. Shah^10^, David Maag^10^

^1^Department of Children and Adolescents, University Hospital Frankfurt, Goethe University Frankfurt, Frankfurt, Germany; ^2^Allergy and Immunology, Department of Medicine, Massachusetts General Hospital, Boston, MA, USA; ^3^Amsterdam Cardiovascular Sciences, Amsterdam University Medical Center, University of Amsterdam, Amsterdam, The Netherlands; ^4^Allergy, Asthma, and Immunology Division, Department of Medicine, Pediatrics, OB/Gyn and Biomedical Sciences, Penn State University, Hershey, PA, USA and Vinmec International Hospital, Times City, Hanoi, Vietnam; ^5^Hungarian Angioedema Center, Department of Internal Medicine and Haematology, Semmelweis University, Budapest, Hungary; ^6^Department of Immunology, Auckland City Hospital, and the Department of Medicine, University of Auckland, Auckland, New Zealand; ^7^Allergy & Asthma Specialists of Dallas and University of Texas Southwestern Medical School, Dallas, TX, USA; ^8^The Institute of Allergology, Charité-Universitätsmedizin Berlin, Freie Universität Berlin and Humboldt-Universität zu Berlin, and Immunology and Allergology, Fraunhofer Institute for Translational Medicine and Pharmacology ITMP, Berlin, Germany; ^9^Division of Allergy & Immunology, University of California, San Diego, San Diego, CA, USA; ^10^Intellia Therapeutics, Cambridge, MA, USA

Allergy, Asthma & Clinical Immunology 2026, 22(Suppl 1):O-31

**Background:** Hereditary angioedema (HAE), a rare genetic disorder, requires lifelong treatment to manage symptoms. HAE manifests as severe and unpredictable swelling attacks through dysregulated bradykinin production by kallikrein. NTLA-2002 is an investigational, in vivo CRISPR-based therapy designed to rebalance the kallikrein-kinin system by permanently editing the *KLKB1* gene to prevent attacks after a single administration. In phase 2 (NCT05120830), the mean difference in monthly attack rate (weeks 5–16) with NTLA-2002 25 mg (n = 10) and 50 mg (n = 11) vs placebo (n = 6) was -80% and -81%. In NTLA-2002–treated participants (N = 21), all adverse events were grade 1/2, most commonly headache (38%), fatigue (29%), and nasopharyngitis (29%). Eight of 11 patients treated with NTLA-2002 50 mg were attack-free through latest assessment (maximum follow-up, 11.5 months), leading to selection of this dose for further investigation [1]. HAELO is a phase 3, global, multicentre, randomised, double-blind, placebo-controlled study evaluating efficacy and safety of NTLA-2002 in participants with HAE (NCT06634420).

**Methods:** Participants (N = 60) aged ≥ 18 years with HAE-C1-INH-Type1 or -Type2 with ≥ 2 investigator-confirmed HAE attacks during the 8-week Run-in Period will be randomised 2:1 to receive a single intravenous infusion of NTLA-2002 50 mg or placebo, stratified by number of investigator-confirmed HAE attacks per month (> 3 vs ≤ 3) at baseline. Participants must refrain from using long-term prophylaxis (LTP) during the Run-in Period through the end of the Primary Observation Period (week 28) and have access to on-demand treatments (ODTs). After week 28, participants may elect to receive a blinded, single intravenous infusion of the opposite treatment; all will enter long-term observation (76 weeks; 104 weeks total post randomisation). The primary endpoint is time-normalised number of investigator-confirmed HAE attacks (weeks 5–28). Key secondary endpoints are time-normalised number of investigator-confirmed attacks requiring ODT (weeks 5–28); time-normalised number of moderate or severe investigator-confirmed attacks (weeks 5–28); investigator-confirmed HAE attack-free status (weeks 5–28); and change from baseline to week 28 in quality of life (QoL), measured by the Angioedema QoL Questionnaire (AE-QoL) total score. Other secondary endpoints are responder status (weeks 5–28; 1–28); LTP-free status (percent LTP-free; weeks 5–104); HAE treatment-free status (percent LTP- and ODT-free; weeks 5–104); complete responder status (zero investigator-confirmed attacks with no LTP; weeks 5–104); reduction in time-normalised investigator-confirmed attacks in placebo-randomised participants after crossover (weeks 33–104 vs screening through week 28); and key secondary endpoints measured from weeks 1–28. Safety and change from baseline in total plasma kallikrein will be assessed. HAELO is currently enrolling.

**Results:** NA.

**Conclusion:** NA.

*Acknowledgements:* *This study is funded by Intellia Therapeutics.*

*Trial registration:* *ClinicalTrials.gov NCT06634420*

Reference:

[1]Cohn DM, Gurugama P, Magerl M, et al. CRISPR-Based Therapy for Hereditary Angioedema. N Engl J Med. 2025;392(5):458-467.

## O-32

### Anxiety associated with hereditary angioedema attacks: Results from the phase 3 KONFIDENT trial of oral sebetralstat

Timothy J. Craig^1^,*, Emel Aygören-Pürsün^2^, Jonathan A. Bernstein^3^, Paula J. Busse^4^, Teresa Caballero^5^, Danny M. Cohn^6^, Mar Guilarte^7^, Henriette Farkas^8^, Douglas H. Jones^9^, Sorena Kiani-Alikhan^10^, Michael E. Manning^11^, Marcus Maurer^12^, Marc A. Riedl^13^, Sinisa Savic^14^, H. James Wedner^15^, Patrick F. K. Yong^16^, Andrea Zanichelli^17,18^, Erik Hansen^19^, James Hao^19^, Michael D. Smith^19^, Christopher M. Yea^19^, Paul K. Audhya^19^, William R. Lumry^20^

^1^Departments of Medicine, Pediatrics, Ob/Gyn MFM and Biomedical Sciences, Pennsylvania State University, Hershey, PA, USA; Vinmec International Hospital, Times City, Hanoi, Vietnam; ^2^University Hospital Frankfurt, Goethe University Frankfurt, Frankfurt, Germany; ^3^University of Cincinnati College of Medicine, and Bernstein Clinical Research Center, Cincinnati, OH, USA; ^4^Department of Medicine, Icahn School of Medicine at Mount Sinai School of Medicine, New York, NY, USA; ^5^Servicio de Alergia, Hospital La Paz Health Research Institute, Biomedical Research Network on Rare Diseases, Madrid, Spain; ^6^Amsterdam University Medical Center, University of Amsterdam, Amsterdam, The Netherlands; ^7^Department of Allergy, Hospital Universitari Vall d'Hebron, Vall d’Hebron Research Institute (VHIR), Barcelona, Spain; ^8^Hungarian Angioedema Center of Reference and Excellence, Semmelweis University, Budapest, Hungary; ^9^Rocky Mountain Allergy, Asthma, and Immunology, Layton, UT, USA; ^10^Division of Infection and Immunity, University College London, London, UK; ^11^Medical Research of Arizona, Scottsdale, AZ, USA; and University of Arizona College of Medicine-Phoenix, Phoenix, AZ, USA; ^12^Institute of Allergology, Charité-Universitätsmedizin Berlin, corporate member of Freie Universitätsmedizin Berlin and Humboldt-Universität zu Berlin, Berlin, Germany, and Fraunhofer Institute for Translational Medicine and Pharmacology ITMP, Immunology and Allergology, Berlin, Germany; ^13^University of California, San Diego, La Jolla, CA, USA; ^14^The Leeds Institute of Rheumatic and Musculoskeletal Medicine, University of Leeds, Leeds, UK; ^15^John T. Milliken Department of Medicine, Washington University School of Medicine, St. Louis, MO, USA; ^16^Department of Immunology, Frimley Health NHS Foundation Trust, Frimley, UK; ^17^Operative Unit of Medicine, Angioedema Center, IRCCS Policlinico San Donato, San Donato Milanese, Milan, Italy; ^18^Department of Biomedical Science for Health, University of Milan, Milan, Italy; ^19^KalVista Pharmaceuticals, Salisbury, United Kingdom, and Cambridge, MA, USA; ^20^AARA Research Center, Dallas, TX, USA

*tcraig@pennstatehealth.psu.edu

Allergy, Asthma & Clinical Immunology 2026, 22(Suppl 1):O-32

**Background:** People living with HAE experience unpredictable, painful, and potentially life-threatening attacks that are associated with substantial psychological burden. A significant component of psychological burden is anxiety, including that related to the need to inject or infuse on-demand treatment. This analysis assessed the impact of sebetralstat, an orally administered on-demand therapy, on remnant anxiety in the phase 3 KONFIDENT trial (NCT05259917).

**Materials and Methods:** In KONFIDENT, participants (≥ 12 years) with HAE-C1-INH treated ≤ 3 attacks with oral sebetralstat 300 mg, 600 mg, or placebo. Participants completed a Modified Generalized Anxiety Numeric Rating Scale (GA-NRS) from 0 (not at all anxious) to 10 (extremely anxious) at attack onset and for 24 h after treatment. Pearson correlation was used to determine the coefficients between GA-NRS and baseline demographics and attack characteristics. Cumulative GA-NRS was calculated as the area under the curve over 12 h (AUC0-12) or 24 h (AUC0-24) from administration. Least squares mean (LSM) changes in GA-NRS from baseline through 12 h post-baseline were calculated. Cohen’s kappa analysis was used to determine agreement of a ≥ 2-point reduction in GA-NRS within 12 h, with achievement of beginning of symptom relief within 12 h (primary endpoint).

**Results:** Of 264 treated attacks, 250 (sebetralstat 300 mg: 83; sebetralstat 600 mg: 87; placebo: 80) included GA-NRS records. Median baseline GA-NRS was 3.0. Among baseline variables, severity of the attack (P = 0.0030 for moderate and P < 0.0001 for severe/very severe versus mild), time since HAE diagnosis (P = 0.0014 for shorter versus longer), and sex (P = 0.0034 for female versus male) correlated with baseline anxiety. AUC0-12 and AUC0-24 were significantly reduced with sebetralstat 300 mg (P = 0.004 and P = 0.022) and 600 mg (P = 0.0008 and P = 0.0012) versus placebo. For participants with moderate-to-extreme anxiety (4–10; median 5.0 for 300 mg; 6.0 for 600 mg and placebo), LSM change from baseline (95% CI) at 4 h was − 2.8 (− 3.6, − 1.9) for each sebetralstat group and − 1.3 (− 2.2, − 0.4) for placebo and at 12 h was − 3.5 (− 4.3, − 2.6) with sebetralstat 300 mg, − 4.3 (− 5.2, − 3.5) with 600 mg, and − 1.7 (− 2.6, − 0.8) with placebo. Participants who had attacks achieving beginning of symptom relief earlier (within 12 h) had a better chance to reach meaningful reductions in anxiety (≥ 2-points) (kappa: 0.47).

**Conclusions:** In KONFIDENT, anxiety was rated as moderate to extreme at the time of treatment in > 40% of attacks. In the absence of injectables, remnant anxiety was correlated with baseline attack severity. Meaningful anxiety reductions were associated with earlier achievement of beginning of symptom relief after oral sebetralstat administration.

## O-33

### Long-term safety and efficacy of oral deucrictibant for prophylaxis in hereditary angioedema: Data snapshot results of the CHAPTER-1 open-label extension study

Emel Aygören-Pürsün^1^, John Anderson^2^, Francesco Arcoleo^3^, Mauro Cancian^4^, Hugo Chapdelaine^5^, Niall Conlon^6^, Efrem Eren^7^, Mark Gompels^8^, Sofia Grigoriadou^9^, Maria D. Guarino^10^, Padmalal Gurugama^11^, Sorena Kiani-Alikhan^12^, Tamar Kinaciyan^13^, Markus Magerl^14,15^, Michael E. Manning^16^, Marcin Stobiecki^17^, Michael D. Tarzi^18^, Anna Valerieva^19^, H. James Wedner^20^, William H. Yang^21^, Andrea Zanichelli^22,23^, Rafael Crabbé^24^, Susan Mulders^25^, Jonathan Levy^26^, Ulrich Freudensprung^27^, Umar Katbeh^27^, Jochen Knolle^28^, Anne Lesage^29^, Peng Lu^26^, Marc A. Riedl^30^

^1^Goethe University Frankfurt, Frankfurt, Germany; ^2^AllerVie Health, Birmingham, AL, USA; ^3^UOC di Patologia Clinica e Immunologia, Palermo, Italy; ^4^University Hospital of Padua, Padua, Italy; ^5^Université de Montréal, Montréal, QC, Canada; ^6^St. James's Hospital and Trinity College, Dublin, Ireland; ^7^University Hospital Southampton NHS Foundation Trust, Southampton, UK; ^8^North Bristol NHS Trust, Bristol, UK; ^9^Barts Health NHS Trust, London, UK; ^10^Ospedale di Civitanova Marche, Civitanova Marche, Italy; ^11^Cambridge University Hospitals NHS Foundation Trust, Cambridge, UK; ^12^Royal Free London NHS Foundation Trust, London, UK; ^13^Medical University of Vienna, Vienna, Austria; ^14^Charité – Universitätsmedizin Berlin, Berlin, Germany; ^15^Fraunhofer Institute for Translational Medicine and Pharmacology, Berlin, Germany; ^16^Allergy, Asthma and Immunology Associates, Ltd., Scottsdale, AZ, USA; ^17^Jagiellonian University Medical College, Krakow, Poland; ^18^University Hospitals Sussex NHS Foundation Trust, Brighton, UK; ^19^Medical University of Sofia, Sofia, Bulgaria; ^20^Washington University School of Medicine, St Louis, MO, USA; ^21^Ottawa Allergy Research Corporation, University of Ottawa, Ottawa, ON, Canada; ^22^Universita degli Studi di Milano, Milan, Italy; ^23^I.R.C.C.S., UO Medicina, Milan, Italy; ^24^RC Consultancy, Bassins, Switzerland; ^25^Mulders Clinical Consulting, Groesbeek, The Netherlands; ^26^Pharvaris Inc., Lexington, MA, USA; ^27^Pharvaris GmbH, Zug, Switzerland; ^28^JCK Consult, Frankfurt, Germany; ^29^GrayMatters Consulting, Schilde, Belgium; ^30^University of California San Diego, La Jolla, CA, USA

Allergy, Asthma & Clinical Immunology 2026, 22(Suppl 1):O-33

**Rationale**: Deucrictibant is a selective, orally administered bradykinin B2 receptor antagonist that is under development for prophylactic and on-demand treatment of hereditary angioedema (HAE) attacks. The two-part, Phase 2 CHAPTER-1 study (NCT05047185) evaluates the efficacy and safety of deucrictibant for long-term prophylaxis of HAE attacks. The randomized, double-blind, placebo-controlled, controlled trial period (part 1) is complete and an open-label extension (OLE; part 2) is currently ongoing. In part 1, deucrictibant 40 mg/day reduced the attack rate by 84.5% (*P* = 0.0008) vs placebo during 12 weeks of treatment, and was well tolerated.

**Methods:** CHAPTER-1 enrolled 34 eligible participants who were aged ≥ 18 and ≤ 75 years, diagnosed with HAE-1/2, and had experienced either ≥ 3 attacks within 3 months prior to screening or ≥ 2 attacks during screening (up to 8 weeks). At screening, no participants were receiving prophylactic treatments. Thirty participants completed part 1, during which they received deucrictibant 20 mg/day (n = 11), 40 mg/day (n = 10), or placebo (n = 9) for 12 weeks. All thirty participants continued into the ongoing OLE and received treatment with deucrictibant 40 mg/day.

**Results:** This data snapshot (cutoff: 10 June 2024) included 30 participants who received deucrictibant 40 mg/day for a mean treatment duration of 12.8 months (maximum ~ 20 months) in the OLE. Participants were 60% female and had a mean age of 39.1 years at part 1 baseline. The safety analysis showed no treatment-related serious treatment emergent adverse events (TEAEs), no TEAEs leading to study drug discontinuation, and that deucrictibant was well tolerated. At study baseline, participants had a mean (standard deviation) attack rate of 2.18 (1.35) and a least squares mean (LSM) attack rate of 0.15 (standard error [SE] 0.05) in the OLE. The attack rate in the OLE was low irrespective of participants’ attack rate at study baseline; 0.10 and 0.19 for participants with ≥ 1 to < 2 attacks per month and ≥ 2 attacks per month at study baseline, respectively. During the OLE, the LSM (SE) monthly rates of “moderate and severe” attacks and attacks treated with on-demand medication was 0.07 (0.03) and 0.07 (0.02), respectively. The median proportion of days with HAE symptoms in the OLE was 0.0%.

**Conclusions:** These data from the ongoing CHAPTER-1 OLE extend the findings of part 1 and provide additional evidence on the long-term safety and efficacy of deucrictibant treatment for prophylaxis of HAE attacks.

## O-34

### Long-term safety and efficacy of oral deucrictibant for treatment of hereditary angioedema attacks: Results of the RAPIDe-2 extension study

Marc A. Riedl^1^, John Anderson^2^, Emel Aygören-Pürsün^3^, Laurence Bouillet^4^, Hugo Chapdelaine^5^, Henriette Farkas^6^, Delphine Gobert^7^, Roman Hakl^8^, Joshua S. Jacobs^9^, Ramón Lleonart^10^, Michael E. Manning^11^, Avner Reshef^12^, Giuseppe Spadaro^13^, Maria Staevska^14^, Marcin Stobiecki^15^, Giorgio Giannattasio^16^, Yumeng Li^17^, Peng Lu^17^, Justin Sun^17^, Ming Yu^17^, Anna Valerieva^14^

^1^University of California San Diego, La Jolla, CA, USA; ^2^AllerVie Health, Birmingham, AL, USA;^3^University Hospital Frankfurt, Goethe University Frankfurt, Frankfurt, Germany; ^4^Grenoble Alpes University, Grenoble, France; ^5^CHU de Montréal, Université de Montréal, Montréal, QC, Canada; ^6^Semmelweis University, Budapest, Hungary; ^7^Sorbonne Université, AP-HP, Hôpital Saint-Antoine, Paris, France; ^8^Masaryk University, Brno, Czech Republic; ^9^Allergy and Asthma Clinical Research, Walnut Creek, CA, USA; ^10^Bellvitge University Hospital, L'Hospitalet de Llobregat, Barcelona, Spain; ^11^Allergy, Asthma and Immunology Associates, Ltd., Scottsdale, AZ, USA; ^12^Barzilai University Hospital, Ashkelon, Israel; ^13^University of Naples Federico II, Napoli, Italy; ^14^Medical University of Sofia, Sofia, Bulgaria; ^15^Jagiellonian University Medical College, Krakow, Poland; ^16^Pharvaris GmbH, Zug, Switzerland; ^17^Pharvaris Inc., Lexington, MA, USA

Allergy, Asthma & Clinical Immunology 2026, 22(Suppl 1):O-34

**Rationale:** Hereditary angioedema (HAE) attacks are caused by excess bradykinin activating bradykinin B2 receptors. Deucrictibant is a selective, orally administered bradykinin B2 receptor antagonist under development for prophylactic and on-demand treatment of HAE attacks. In the Phase 2, placebo-controlled RAPIDe-1 trial (NCT04618211), deucrictibant immediate-release (IR) capsule showed rapid onset of action, reduced times to symptom relief and to resolution of HAE attacks, and treatment was well tolerated.

**Methods:** RAPIDe-2 (NCT05396105) is an ongoing, two-part, Phase 2/3 extension study evaluating the long-term safety and efficacy of deucrictibant IR capsule for on-demand treatment of repeat HAE attacks. RAPIDe-2 Part A enrolled adult participants who completed RAPIDe-1. Participants continued to self-administer the same double-blinded dose of deucrictibant IR capsule (10 mg, 20 mg, or 30 mg) received during RAPIDe-1 to treat qualifying attacks including non-severe laryngeal attacks presenting without breathing difficulties. Part A is now complete and final topline data are being analyzed.

**Results:** A Part A data snapshot (cutoff: 01 March 2024) included 265 attacks treated with deucrictibant IR capsule by 17 participants. Due to dose-blinding, we report combined dose group results. In the safety analysis, deucrictibant IR capsule was well tolerated with no treatment-related treatment emergent adverse events (TEAEs) and no TEAEs leading to treatment discontinuation. As measured using the Patient Global Impression of Change (PGI-C), the median (95% CIs) time to onset of symptom relief was 1.1 h (1.0–1.2) and to substantial symptom relief was 2.7 h (2.1–2.9). Substantial symptom relief was achieved by 12 h in 96.2% of attacks. Using the PGI of Severity (PGI-S), the median time to attack severity reduction was 2.6 h (2.0–2.9) and to complete attack resolution was 11.5 h (11.0–13.0). Complete resolution within 24 h was achieved in 85.8% of attacks, with 90.2% of these attacks achieving this milestone with a single dose of deucrictibant IR capsule. Rescue medication was used in 4/265 (1.5%) attacks after deucrictibant.

**Conclusions:** Results of the ongoing RAPIDe-2 extension study provide further evidence on the long-term safety and efficacy of deucrictibant IR capsule for on-demand treatment of repeat HAE attacks. In this data snapshot, the median time to onset of symptom relief was 1.1 h with deucrictibant IR capsule treatment and to complete attack resolution was 11.5 h, with the majority of attacks being treated with one dose. Final Part A topline data will be presented.

## O-35

### ADX-324, A Q6-Month SC Investigational siRNA Targeting Prekallikrein for HAE Attack Prevention

Gourab Datta, Jia Hu, Zhen Li, Rui Zui, Robert Macleod, Christopher Storgard

ADARx Pharmaceuticals, San Diego, California, USA

Allergy, Asthma & Clinical Immunology 2026, 22(Suppl 1):O-35

**Rationale:** For hereditary angioedema (HAE), the treatment goal extends beyond merely reducing the frequency of attacks; it is crucial to reduce treatment burden and provide patients with the highest likelihood of achieving attack-free living. ADX-324 is an investigational siRNA that strongly inhibits the production of prekallikrein (PKK), reducing bradykinin generation, preventing HAE attacks. A phase 3 study with ADX-324 as a prophylactic HAE therapy is being initiated with subcutaneous (SC) injection of 300 mg per dose every 6 months.

**Methods:** A Phase 1, healthy volunteer placebo-controlled single dose escalation study of ADX-324 in 6 cohorts (0.4 mg/kg to 6 mg/kg) and 1 cohort administered 2 doses of 140 mg ADX-324 or placebo 3 months apart, and an open-label Phase 2a study in HAE patients with ADX-324 administered SC every 6 months was conducted (Study ADX-324–101, NCT05691361). PKK levels and safety assessments were performed for up to 1 year in Phase 1 and are ongoing in Phase 2a. Three HAE patients have been dosed with ADX-324 at 2 mg/kg (n = 1, approximately 125 mg) and 245 mg (n = 2). Results are shared here from data-cut as of December 5th, 2024.

**Results:** In the placebo-controlled single dose escalation study of ADX-324, a strong dose-dependent PKK-KK reduction was observed. A single SC injection of ADX-324 of 300 mg (± 25 mg) achieved a mean (SEM) PKK-KK reduction from baseline of > 90% at nadir, and > 80% reduction % maintained out to Day 169 (Week 25), This data supports that Q6M dosing of ADX-324 at 300 mg dose level is anticipated to control HAE Furthermore, in a phase 2a, dose range finding study, one HAE patient was dosed at 122 mg and two patients were dosed at 245 mg. Results in these patients demonstrated that patients PKK-KK reductions mirrored those in healthy subjects dosed at the same dose levels. This supports the anticipated PKK-KK reduction of > 80% through 6 months in HAE patients at 300 mg dose. The patients’ data also supports that PKK-KK response can be used to predict HAE attacks and PKK-KK reductions > 70% are likely to prevent HAE attacks.

PKK ADX-324 was well-tolerated with headache and injection-site reaction being the most common AEs in Phase 1 portion (28.9%). No related SAEs or withdrawal due to AEs were reported.

**Conclusions: **ADX-324 is an investigational siRNA targeting PKK in Phase 3 development for HAE-attack prevention. A single subcutaneous injection of ADX-324 at 300 mg provides deep and durable PKK suppression achieving greater than 90% PKK reduction that is maintained below -80% through 6-months.

## O-36

### Transient hepatitis after discontinuation of danazol in patients with hereditary angioedema due to C1-inhibitor deficiency: A case series

Mats de Lange MD^1^, R. Bart Takkenberg MD PhD^2^, Danny M. Cohn MD PhD^1^

^1^Amsterdam UMC, Department of Vascular Medicine, Amsterdam Cardiovascular Sciences, University of Amsterdam, Amsterdam, The Netherlands; ^2^Amsterdam UMC, Department of Gastroenterology and Hepatology, Amsterdam Gastroenterology Endocrinology Metabolism, University of Amsterdam, Amsterdam, The Netherlands

Allergy, Asthma & Clinical Immunology 2026, 22(Suppl 1):O-36

**Background:** Attenuated androgens, such as danazol and stanozolol, have been frequently used as long-term and short-term prophylaxis for hereditary angioedema, but recent guidelines recommend these drugs as second-line therapy only. Discontinuation of long-term danazol use can cause transient hepatitis, a phenomenon not previously described in medical literature.

**Methods:** After the initial observation of transient hepatitis following discontinuation of long-term danazol use in one patient with hereditary angioedema, we prospectively collected clinical and laboratory data in consecutive patients that permanently discontinued danazol use in 2024.

**Results:** A total of four patients with hereditary angioedema discontinued danazol abruptly and all had transaminase elevations after discontinuation. Three of these patients (75%) were female. The median age at discontinuation was 55.5 years (range 51–65). Patients had used danazol for a median of 28 years (range 25–30), with dosages ranging from 300 to 1400 mg per week. All patients discontinued danazol to transition to modern long-term prophylactic treatments. Routine laboratory and ultrasound checks during danazol treatment had been unremarkable and none of the patients used concomitant hepatotoxic medication or consumed excessive amounts of alcohol. Alanine transaminase (ALT) and aspartate transaminase (AST) levels increased within four weeks of discontinuation of danazol, with higher elevations in ALT than AST levels. Peak ALT values ranged from 104 to 337 U/L (reference < 34 U/L), and peak AST values ranged from 53 to 101 U/L (reference < 40 U/L). There were no symptomatic signs of hepatitis or liver-failure, such as jaundice or encephalopathy. Oher causes of hepatitis, including viral infections, auto-immune hepatitis, hemochromatosis and Wilson’s disease were ruled out. A liver biopsy, performed six weeks after discontinuation of danazol in one patient, showed dysfunctional hepatic microcirculation without increased fibrosis and a notable clearance reaction with activated Kupffer cells. All ALT and AST values returned to or below the upper limit of normal within 17 weeks, without medical intervention.

**Conclusion:** Abrupt discontinuation of long-term danazol use can cause a transient, self-limiting hepatitis with normalization within 17 weeks, presumably due to altered hepatic microcirculation.

## O-37

### Very Long-Term Efficacy of Oral Berotralstat-LTP in hereditary angioedema patients

Tamar Kinaciyan^1^, Amélie Hampel^1^, Elias Marquart^1^, Max Ross^2^, Sven Pohl^2^

^1^Department of Dermatology, Medical University of Vienna, Vienna, Austria; ^2^BioCryst Pharma Deutschland GmbH, Munich, Germany

Allergy, Asthma & Clinical Immunology 2026, 22(Suppl 1):O-37

**Background:** Hereditary angioedema, HAE, is a rare inherited disease with unpredictable, painful and potentially life-threatening swelling attacks, high disease burden and massive impairment of quality of life. Berotralstat is an oral, selective kallikrein inhibiting small molecule approved for long-term prophylaxis, LTP, in HAE-patients. We report a case series of 4 patients who are successfully treated with Berotralstat since mid of 2018 (cutoff: 20 December 2024).

**Methods:** Four patients (2 ladies) with a median age of 54 years suffering from HAE type I from Vienna were treated with Berotralstat 150 mg capsule once daily. We evaluated the effectiveness of Berotralstat-LTP on the patient-reported occurrence of HAE attacks, their severity and number of attacks where on-demand treatment was used, collected at every clinical visit. In addition, we evaluated safety, tolerability and treatment emergent adverse events (TEAEs).

**Results:** In 2017, the year preceding the initiation of Berotralstat-LTP, patients experienced between 2 and 4 mild to severe HAE attacks per month. Specifically, Patients 1 and 3 reported two attacks per month, Patient 2 experienced three attacks per month, and Patient 4 had at least one attack per month. This resulted in a total of 96 attacks over 12 months, with a mean of 2 attacks per patient per month. Over a follow-up period of 66 months (5.5 years) under Berotralstat-LTP, the mean attack rate was significantly reduced. Patient 1 experienced 0.29 attacks per month (19 attacks in total), while Patient 2 had 0.26 attacks per month (17 attacks in total). In contrast, Patient 3 reported only 0.06 attacks per month (4 attacks in total), and Patient 4 experienced 0.1 attacks per month (7 attacks in total). This corresponds to an overall mean attack reduction of 90%. Additionally, attack severity decreased to mild-to-moderate, with on-demand treatment required for only 35/47 attacks (74%). Attack-free periods ranged from 120 to 1,304 days (~ 43 months). Berotralstat was well tolerated, with no safety concerns or treatment-emergent adverse events (TEAEs) reported.

**Conclusion:** To the best of our knowledge, this report presents the longest follow-up period of HAE patients on Berotralstat-LTP. Throughout the entire monitoring period, we observed sustained efficacy of Berotralstat, with prolonged attack-free periods and an overall mean attack reduction of approximately 90% in our patients.

## O-38

### Tolerability and effectiveness of berotralstat for prophylaxis in adolescents with hereditary angioedema: Subgroup analysis of the Berolife study

Mélisande Bourgoin Heck^1^, Yann Ollivier^2^, Fabien Pontille^3^, Hervé Maillard^4^, Delphine Gobert^5^, Douglas Johnston^6^, Mona Villedieu^6^, Nicolas Lemaire^7^, Laure Carriat^8^, Isabelle Boccon-Gibod^9^

^1^French National Reference Center for Angioedema (CREAK), Department of Internal Medicine, Saint Antoine Hospital, and Allergy Department, Trousseau Hospital, Sorbonne Université, Paris, France; ^2^Department of Allergy, Caen University Hospital, Caen, France; ^3^Department of Internal Medicine, Nancy University Hospital, Nancy, France; ^4^Department of Dermatology, Le Mans Hospital Center, Le Mans, France; ^5^Sorbonne Université, French National Reference Center for Angioedema (CREAK), Department of Internal Medicine, Saint Antoine Hospital, Paris 6 University, Paris, France; ^6^BioCryst Pharmaceuticals, Inc., Durham, NC, USA; ^7^Axonal-Biostatem, Paris, France; ^8^BioCryst Pharmaceuticals France, Medical Affairs Department, Boulogne-Billancourt, France; ^9^French National Reference Center for Angioedema (CREAK), Department of Internal Medicine, Grenoble Alpes University Hospital, Grenoble, France

Allergy, Asthma & Clinical Immunology 2026, 22(Suppl 1):O-38

**Rationale:** Berotralstat is a first-line, once-daily oral prophylactic treatment for hereditary angioedema (HAE), a rare genetic disorder. Outcomes on the use of berotralstat in adolescents are limited. The Berolife study is a French observational study designed to assess the real-world tolerability and effectiveness of berotralstat.

**Methods:** Berolife was an open-label, multicentre, longitudinal study conducted in France between September 2021 and January 2024. The study followed an entirely observational design, with no mandatory visits, and patient follow-up was conducted according to routine clinical practise in participating centres. The primary objective was to evaluate the tolerability of berotralstat. Secondary objectives included characterising the patient population and assessing the effectiveness of berotralstat. A subgroup analysis was conducted on the adolescent (12 to 17 years) patient population.

**Results**: A total of 10 adolescents (4 males, 6 females) were enrolled, with a mean age of 15.1 ± 2.4 years and an average disease onset at 7.0 ± 3.5 years old. All patients had HAE-C1-INH-Type1, and 3 had prior long-term prophylaxis (tranexamic acid, n = 2; Lanadelumab, n = 1). The mean treatment duration was 12.6 ± 8.9 months. Seven adverse events (AEs) related to treatment were reported in 5 patients, primarily gastrointestinal disorders such as diarrhoea (2/10) and upper abdominal pain (2/10). Importantly, no serious treatment-related AEs were observed.

Nine adolescents were treated with berotralstat for ≥ 6 months and evaluated at varying follow-up intervals for effectiveness. At 6 months, 6 patients completed a follow-up visit, showing a reduction in mean monthly attack rates (2.43 ± 0.82 at baseline vs. 0.65 ± 0.52). These reductions were also observed at 12 months (n = 4; 2.09 ± 1.42 at baseline vs. 0.60 ± 0.38), and 18 months (n = 3; 2.61 ± 1.24 at baseline vs. 0.58 ± 0.22).

**Conclusions:** In the real world, berotralstat demonstrated a tolerability profile in adolescents consistent with that observed in adults. Additionally, berotralstat effectively reduced attack rates, supporting its role as a valuable treatment for long-term prophylaxis in adolescents with HAE.

*Funding source: BioCryst Pharmaceuticals, Inc.*

## O-39

### Long-term prophylaxis usage for hereditary angioedema by Canadian physicians

Natasha Correa^1,2^,*, Stephen Betschel^1,2^

^1^University of Toronto, Toronto, Ontario, Canada; ^2^Division of Clinical Immunology and Allergy, Department of Medicine, St. Michael's Hospital, Toronto, Ontario, Canada

*natashacorrea@gmail.com

Allergy, Asthma & Clinical Immunology 2026, 22(Suppl 1):O-39

**Background:** Guidelines recommend that physicians consider using long term prophylaxis (LTP) for hereditary angioedema (HAE) patients to prevent HAE attacks and improve quality of life [1]. It is unknown how HAE management practices in Canada have changed over time with the availability of new medications. The objective of our study was to understand use of LTP among Canadian physicians.

**Materials and Methods:** We conducted a survey of HAE treating physicians in Canada in 2017 and 2024. Participants were recruited from clinical organizations that include physicians who treat HAE. Participants were asked about their experiences with using LTP.

**Results:** Thirty-four physicians completed the survey in 2017 and 39 in 2024. Physicians reported a similar proportion of patients on LTP in 2017 and 2024 for HAE type 1 and 2 (53.9% vs. 57.5%) and HAE with normal C1-inhibitor (nC1-INH) (53.4% vs. 53.2%). In 2017, most physicians (93.5%) used intravenous (IV) plasma-derived C1 esterase inhibitor (pdC1-INH) for LTP in all types of HAE, followed by danazol (41.9%), and tranexamic acid (TXA) (19.4%). In 2024, for patients with type 1 and type 2 HAE, 78.4% of physicians used Lanadelumab, 64.9% used berotralstat, 59.5% used IV pdC1-INH, 48.6% used subcutaneous pdC1-INH, 35.1% used TXA and 13.5% used danazol. In 2024, for patients with HAE nC1-INH, 55.9% of physicians used berotralstat, 55.9% used IV pdC1-INH, 38.2% used subcutaneous pdC1-INH, 35.3% used TXA, 20.6% used Lanadelumab and 8.8% used danazol (Fig. 1). Physicians with greater than 10 patients with HAE types 1 and 2 were more likely to prescribe IV pdC1-INH (81% vs. 9%), Lanadelumab (88% vs. 55%) and berotralstat (85% vs. 18%) than physicians with 10 or fewer (p < 0.05). In 2024, factors ranked by physicians as important in deciding to prescribe LTP were frequency of attacks, severity of attacks, and normalization of patients’ lives. The most important non-efficacy factors influencing physician choice of LTP were route of administration (28%), side effects (28%), and access to medication (25%). The most common barriers encountered by physicians in prescribing LTP were administrative challenges (61%), lack of infusion centers (44%) and patients' ability to administer parenteral medications (42%).

**Fig. 1:**
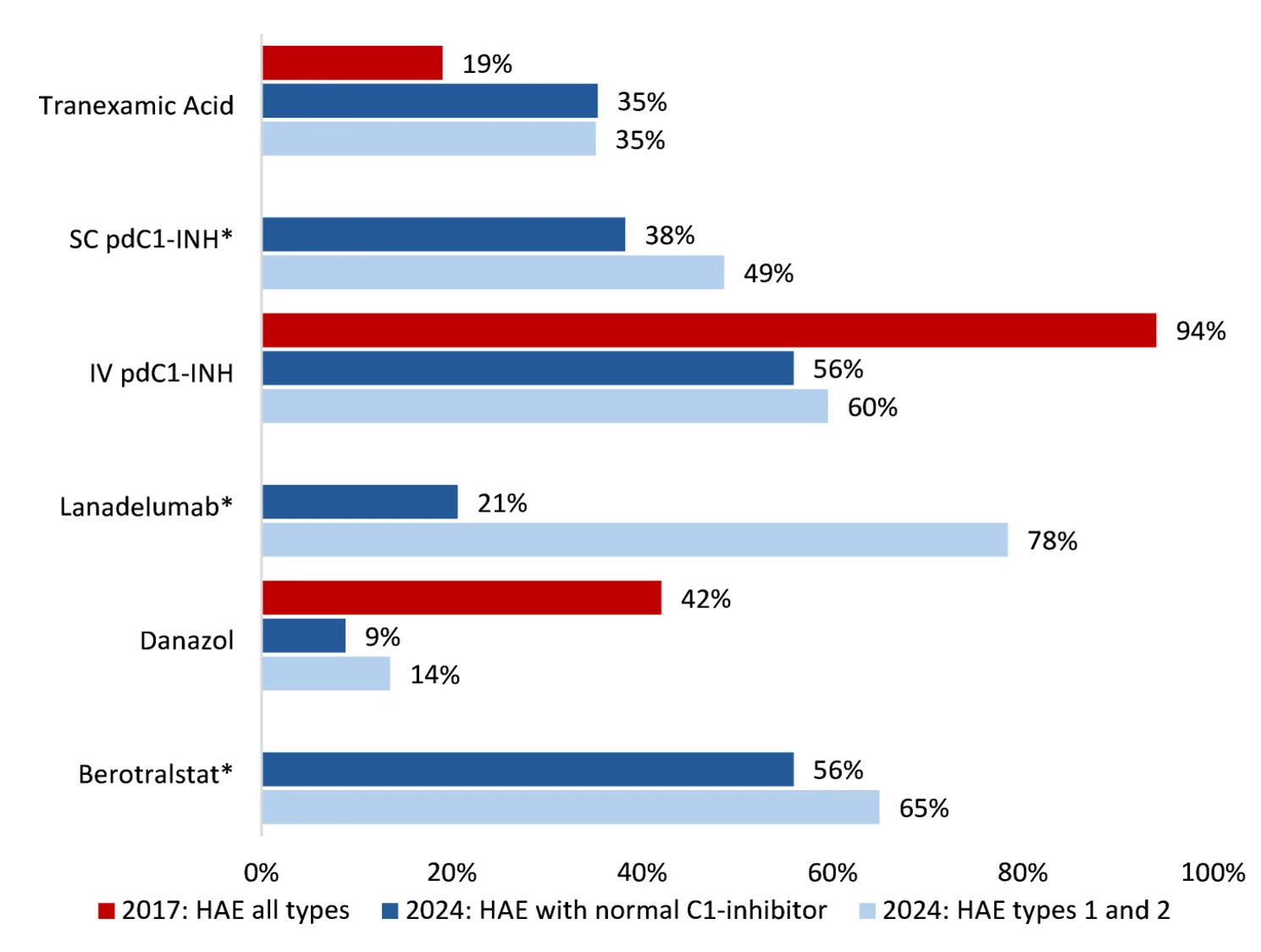
Clinician-reported treatments used for long-term prophylaxis. pdC1-INH, plasma-derived C1 esterase inhibitor; HAE, hereditary angioedema; IV, intravenous; SC, subcutaneous. *No data for SC pdC1-INH, Lanadelumab and berotralstat in 2017

**Conclusions:** Physicians prioritized route of administration and side effects when choosing LTP for HAE. Despite the overall stability in the proportion of patients on LTP, HAE management among Canadian physicians has shifted towards increased use of Lanadelumab and berotralstat and a decline in use of Danazol and IV pdC1-INH.

References:

[1] Betschel S, Badiou J, Binkley K, Borici-Mazi R, Hébert J, Kanani A, et al. The International/Canadian Hereditary Angioedema Guideline. Allergy, Asthma & Clinical Immunology. 2019 Nov 25;15(1).

## O-40

### Berotralstat for the prevention of hereditary angioedema attacks: Results from the Italian expanded access programme

Paola Triggianese^1^, Luisa Brussino^2^, Maria Domenica Guarino^3^, Pietro Accardo^4^, Fabio Buelli^5^, Marica Giliberti^6^, Francesca Perego^7^, Valentino Polo^5^, Riccardo Senter^8^, Giuseppe Spadaro^9^, Alessandra Vultaggio^10^, Andrea Zanichelli^11^

^1^Rheumatology, Allergology and Clinical Immunology, Department of "Medicina dei Sistemi", University of Rome Tor Vergata, Rome, Italy; ^2^SSDDU Immunologia Clinica ed Allergologia, AO Mauriziano, Turin, Italy; ^3^Presidio Ospedaliero di Civitanova Marche, Civitanova Marche, Italy; ^4^A.O. Ospedali Riuniti Villa Sofia-Cervello, Palermo, Italia; ^5^BioCryst Pharmaceuticals Italy, Medical Affairs Department, Rome, Italy; ^6^Azienda Ospedaliero-Universitaria “Policlinico Consorziale”, Bari, Italy; ^7^IRCCS Maugeri Milano, Milan, Italy; ^8^Università degli Studi di Padova, Padua, Italy; ^9^Università degli Studi di Napoli “Federico II”, Naples, Italy; ^10^Azienda Ospedaliero Universitaria Careggi, Florence, Italy; ^11^IRCCS Policlinico S. Donato, San Donato Milanese, Italy

Allergy, Asthma & Clinical Immunology 2026, 22(Suppl 1):O-40

**Background:** Berotralstat is a small molecule, oral kallikrein inhibitor, indicated for the long-term prevention (LTP) of attacks in hereditary angioedema (HAE). Here we report outcomes from the Italian cohort of patients enrolled in the berotralstat Expanded Access Programme (EAP) and treated at ITACA network centres. The EAP was established to provide a therapeutic option for patients without alternative treatment options or refused other LTP treatment until berotralstat became commercially available in Italy.

**Patients and Methods:** Patients eligible for the EAP in Italy received berotralstat free of charge until commercial availability and for an additional 12 weeks to ensure treatment continuity. Safety was monitored throughout the study, and outcomes on breakthrough attacks, disease control (AngioEdema Control Test [AECT]) and quality of life (AngioEdema Quality of Life questionnaire [AEQoL]) were collected.

**Results:** The Italian cohort comprised 22 patients who received at least one dose of berotralstat. The population included 36% males and 64% females, with a mean age of 44.8 years. Comorbidities were present in 59% of patients (13/22). The majority had prior experience with LTP, including androgens (32%), Lanadelumab (18%), intravenous C1-inhibitor (9%), and subcutaneous C1-inhibitor (4%). In patients that completed at least 6 months of treatment (n = 14), the median attack rate decreased from 1.00 per month at baseline to 0.67 after 3 months and 0.33 after 6 months with 42% (6/14) achieving an attack-free status at six months. Median AECT scores increased from 11.50 (n = 12) at baseline to 13.00 at 3 months and 16.00 at 6 months (higher scores indicating improved disease control). Median AEQoL total scores (n = 12) decreased from 45.00 at baseline to 40.00 at 3 months and 34.00 at 6 months (lower scores indicating improved QoL). A total of 9 patients discontinued treatment during the EAP due to pregnancy (n = 1, therapy resumed after pregnancy), adverse events (n = 5), and unsatisfactory treatment outcomes (n = 3). Following the EAP, 15 patients transitioned to standard commercially available berotralstat treatment.

**Conclusions:** Treatment with berotralstat led to a consistent low attack rate with improvements in disease control, and a safety profile consistent with that observed in clinical trials.

## O-41

### Real-world effectiveness of Lanadelumab in hereditary angioedema in Slovakia –A 4-year experience

Milos Jesenak^1,2,3^, Katarina Hrubiskova^4^, Otilia Petrovicova^1,2^, Branislav Slenker^1^, Adam Markocsy^1,2^, Anna Bobcakova^1,2,3^, Martina Ondrusova^5,6^

^1^National Centre for Hereditary Angioedema, Department of Pediatrics and Adolescent Medicine, Jessenius Faculty of Medicine in Martin, Comenius University in Bratislava, University Hospital in Martin, Martin, Slovakia; ^2^Institute of Clinical Immunology and Medical Genetics, Jessenius Faculty of Medicine in Martin, Comenius University in Bratislava, University Hospital in Martin, Martin, Slovakia; ^3^National Centre for Hereditary Angioedema, Department of Pulmonology and Phthisiology, Jessenius Faculty of Medicine in Martin, Comenius University in Bratislava, University Hospital in Martin, Martin, Slovakia; ^4^National Centre for Hereditary Angioedema, 5th Department of Internal Medicine, Faculty of Medicine of Comenius University in Bratislava, University Hospital Bratislava, Bratislava, Slovakia; ^5^Pharm-In, Ltd., Bratislava, Slovakia; ^6^Department of Epidemiology, Faculty of Public Health, Slovak Medical University, Bratislava, Slovakia

Allergy, Asthma & Clinical Immunology 2026, 22(Suppl 1):O-41

**Introduction:** Hereditary angioedema (HAE) represents a life-threatening rare form of inborn error of immunity with significant impact on all the domains of quality of life of the affected patients and their families. Innovative therapies are able to dramatically change the clinical course of HAE, improve quality of life and allow the achievement of full clinical control in the majority of the patients. Current prevalence of HAE in Slovakia (5.447 million of citizens) is 1:41 280 and incidence 1:1,360,000 (altogether, 132 living patients). Lanadelumab (LAN) is available and reimbursed since 2021 as a standard-of-care long-term prophylaxis.

**Patients and Methods:** We evaluated the clinical effectiveness and tolerability in a group of HAE patients from Slovakia treated with Lanadelumab after fulfilling the indication criteria for this treatment.

**Results:** 30 patients treated with Lanadelumab were identified (21 females, 70%; median age 41.73 ± 18.67 years; age range 11 – 82 years). Mean number of attacks during the 12 months before the initiation of LAN was 23.73 ± 9.86 (range 9 – 47/year). The majority of the patients was treated with other forms of long-term prophylaxis before LAN – attenuated androgens, tranexamic acid, pdC1-INH concentrate or berotralstat (76.7%). Patients suffered from a broad spectrum of HAE attacks: 93.33% abdominal, 93.33% peripheral, 90% orofacial, 76.67% laryngeal and 63.33% urogenital. 22 patients were treated with LAN for more than 12 months and were used for the further analyses. LAN decreased the mean attack rate from 24.64 ± 8.83 to 1.27 ± 1.45/year and 7 patients remained completely asymptomatic (40.91%). LAN reduced the number of attacks by 94.89% and 51.85% of attacks were of mild intensity. 17 patients were treated for more than 2 years and the proportion of asymptomatic patients increased to 58.82%. Further sub-analyses were performed in 25 patients treated for at least 6 months. The mean monthly frequency of attacks decreased significantly from 1.75 ± 0.75 to 0.09 ± 0.09 (p < 0.001) and moderate-to-severe attacks dropped from 1.05 ± 0.69 to 0.05 ± 0.08 (p < 0.001). LAN decreased the monthly frequency of HAE attacks by 95.13%. LAN treatment was well tolerated, and no serious adverse events were observed. No patient discontinued the treatment.

**Conclusions:** Lanadelumab showed very high real-world effectiveness in a cohort of HAE patients in Slovakia. It significantly decreased not only the mean number of the attacks but also decreased significantly the proportion of moderate-to-severe attacks. LAN represents a true “game-changer” in life of HAE patients.

## O-42

### Harnessing innovation to collect data for Harpe, a dynamic French registry of patients with hereditary angioedema: Results from the single-centre phase

Alexis Bocquet^1^,*, Trevor Stanbury^3^, Carole Requillard^2^, Isabelle Boccon-Gibod^1^, Mélanie Arnaud^1^, Lisa Chutoo^2^, Aliette Poplu^2^, Pierre Rolland^2^, Benoit Bouquillon^3^, Franck Le Ouay^2^, Laurence Bouillet^1^

^1^Internal Medicine Department, Grenoble Alpes University Hospital, Grenoble, France; ^2^Lifen, Paris, France; ^3^Carely, Lille, France

*abocquet@chu-grenoble.fr

Allergy, Asthma & Clinical Immunology 2026, 22(Suppl 1):O-42

**Background:** In rare diseases like hereditary angioedema (HAE), real-world evidence (RWE) is scarce, and patient registries are essential for data collection. Traditional registries are costly and timeconsuming, relying on medical records that need to be regularly updated. For HAE, patients usually have follow-up visits only once or twice a year, resulting in outdated registry data. This is problematic, particularly when numerous novel therapies are being approved and become available.

**Methods:** To address these challenges, the French network for angioedema (CREAK), coordinated by CHU Grenoble Alpes, launched HARPE, a dynamic registry that uses innovation to regularly collect and update patient data. Lifen, an AI-based solution, was employed to automate the extraction and updating of data from medical files. The process was implemented for patients treated at CHU Grenoble Alpes.

**Results:** Lifen identified 265 HAE patients from the CHU Grenoble Alpes medical files: 72 had at least one physical follow-up visit in the last 12 months, and 129 in the last 36 months. For the 129 patients with a visit in the last 36 months, the median age at first attack (known for 56 patients) was 10.0 years (IQR: 4.9–17.1), and the median age at diagnosis (known for 52 patients) was 17.6 years (IQR: 6.7–36.3). The cohort was predominantly female (67.4%). Among them, 92 had type 1 HAE, 6 had type 2, 29 had type 3, and 2 had an unknown type. The median duration of follow-up from diagnosis (known for 51 patients) was 13.69 years (IQR: 8.1–18.6). Thirty-seven patients (28.7%) used on-demand treatment, and 76 (58.9%) used long-term prophylaxis. During the 36 months, 40 patients (31.0%) consulted once, 31 (24.0%) consulted twice, and 58 (45.0%) consulted more than twice. A total of 1013 attacks were reported by 85 patients, with a median of 2 attacks (IQR: 1–5) per patient. Of these attacks, 45.7% were abdominal, 8.8% affected the face, and 23.2% occurred in the extremities. Ten patients were hospitalized or visited the emergency department for at least one attack.

**Conclusion:** Digital innovation, such as AI-based solutions, can be utilized to develop and maintain a dynamic HAE patient registry, ensuring the regular update and enrichment of data. Biocryst provided institutional funding for HARPE.

## O-43

### Turkish National Hereditary Angioedema and Acquired Angioedema Registry, HAE/AAE-TR

E. Nihal Mete Gokmen^1^,*, G. Ozden^2^, E. Karakoç Aydıner^3^, B. Sin^4^, G. Bulut^5^, Ö. Özdemir⁶, R. Tülümen Öztürk⁷, S. Taşyürek⁸, NY. Akçam⁹, I. Bulut^10^, C. Kırmaz^11^, N. Sevimli^12^, H. Senol Duman^13^, FM. Tepetam^10^, E. Topyıldız^13^, S. Cildağ^14^, M. Kılıç^15^, M. Poyraz^16^, A. Burkan Akyıldız^17^, I. Ozanlı^18^, G. Alpagat^19^, H. Aytaç^20^, S. Bayrak^12^, G. Boğatekin^21^, A. Can^22^, F. Gürlek^23^, P. Gökmirza Özdemir^24^, EN. Koç^17^, S. Mersin^25^, I. Yıldırım^24^, F. Genel^26^, M. Keren^10^, C. Orçen^27^, R. Yıldız^28^, M. Özışık^29^, A. Çaymar^30^, AZ. Sin^1^, M. Erkoç^2^, L. Çevirme^2^, E. Yalçın Güngören^33^, E. Karabiber^31^, F. Aytekin^34^, A. Yıldırım^4^, E. Tüsüz Önata^6^, U. Dikici⁶, D. Ediger⁷, A. Bahadir^1^, D. Yavuz^33^, S. Demirsoy^11^, M. Geyik^1^, Ö. Atik^10^, S. Türk Şahin^14^, M. Demir^34^, K. Okan^1^, H. Serpil Akten^1^, C. Tunakan Dalgıç^1^, R.F. Kural^1^, R. Gümüşburun^1^, S. İnan^29^, O. Yıldırım^1^, AO. Özen^3^, S. Barış^3^, S. Bilgiç Eltan^3^, S. Altay^1^, E. Dilek^1^, A. Kılavuz^1^, AM. Ergin^32^, S. Özgül^32^

^1^Ege University Medical Faculty, Izmir, Turkey; ^2^Adana City Training and Research Hospital, Adana, Turkey; ^3^Marmara University Faculty of Medicine, İstanbul, Turkey; ^4^Ankara University, School of Medicine, Ankara, Turkey; ^5^Balıkesir Atatürk City Hospital, Balıkesir, Turkey; ^6^Faculty of Medicine Sakarya University Research and Training Hospital, Sakarya, Turkey; ^7^Bursa Uludag University, Faculty of Medicine, Bursa, Turkey; ^8^Mardin State Hospital, Mardin, Turkey; ^9^Mersin Sehir Eğitim ve Araştırma Hastanesi, Mersin, Turkey; ^10^Health Sciences University Süreyyapaşa Chest Disease and Chest Surgery Training and Research Hospital, İstanbul, Turkey; ^11^Celal Bayar University Medical Faculty, Manisa, Turkey; ^12^Diyarbakır Gazi Yaşargil Eğitim Ve Araştırma Hastanesi, Diyarbakır, Turkey; ^13^Hatay Training and Research Hospital, Hatay, Turkey; ^14^Aydın Adnan Menderes University, Medical Faculty, Aydın, Turkey; ^15^Fırat University Faculty of Medicine, Elâzığ, Turkey; ^16^Mehmet Akif İnan Eğitim ve Araştırma Hastanesi, Şanlıurfa, Turkey; ^17^Gaziantep Sehir Hastanesi, Gaziantep, Turkey; ^18^Kocaeli University Research and Application Hospital, İzmit, Turkey; ^19^Malatya Education and Research Hospital, Malatya, Turkey; ^20^Erzurum Sehir Hastanesi, Erzurum, Turkey; ^21^Sakarya Eğitim ve Araştırma Hastanesi, Sakarya, Turkey; ^22^University of Health Sciences, Van Training and Research Hospital, Van, Turkey; ^23^Health Sciences University, Bursa Medical Faculty, Bursa, Turkey; ^24^Başakşehir Cam and Sakura City Hospital, İstanbul, Turkey; ^25^Muğla Eğitim ve Araştırma Hastanesi, Muğla, Turkey; ^26^B.U. Dr. Behçet Uz Çocuk Hastalıkları Ve Cerrahisi Eğitim ve Araştırma Hastanesi, İzmir, Turkey; ^27^Göztepe Prof. Dr. Süleyman Yalçın Şehir Hastanesi, İstanbul, Turkey; ^28^Şanlıurfa Eyyübiye Eğitim ve Araştırma Hastanesi, Şanlıurfa, Turkey; ^29^İzmir City Hospital, İzmir, Turkey; ^30^Çiğli Training Research Hospital, İzmir, Turkey; ^31^Pendik Research and Training Hospital, İstanbul, Turkey; ^32^Ege University, İzmir, Turkey; ^33^University of Health Sciences Yedikule Chest Disease and Chest Surgery Training and Research Hospital, İstanbul, Turkey; ^34^University of Health Sciences, Kartal Dr Lütfi Kırdar City Hospital, Istanbul, Turkey

*nihal.gokmen@ege.edu.tr

Allergy, Asthma & Clinical Immunology 2026, 22(Suppl 1):O-43

**Background:** Hereditary angioedema (HAE) due to C1-inhibitor (C1-INH) deficiency (HAE-C1-INH) is a rare but potentially life-threatening genetic disorder. In Turkey, its prevalence may be higher due to consanguineous marriages, which increase the inheritance of autosomal dominant conditions. Despite advances in diagnostics, the time from symptom onset to diagnosis often exceeds 20 years, leading to significant morbidity and unnecessary interventions. This delay is mainly due to low disease awareness and frequent misdiagnosis with conditions like allergic angioedema, familial Mediterranean fever, appendicitis, or ovarian cyst rupture. To address this, Turkish National Hereditary Angioedema Registry was developed for systematic patient data collection and improved clinical decision-making.

**Methods:** Turkish Hereditary Angioedema Registry is a role-based web platform with PostgreSQL 12 on Ubuntu 18.04 Linux as the database server and Microsoft.NET Core on Windows Server 2019 as the web server. Authorized physicians input patient data, including demographics, genetic and biochemical test results, family relationships, lifestyle, attack occurrences, and medication adherence. This real-time monitoring tool enhances clinical follow-up and research on hereditary angioedema in Turkey.

**Results:** The HAE/AAE-TR Registry includes data from 450 patients across 55 centers in 25 cities, with 79 registered users contributing. The highest proportion of centers is in Marmara (32.7%), followed by Aegean (20.0%) and Central Anatolia (16.3%). Among the patients, 59.5% are female, and 40.5% are male. The median age of symptom onset is 11 years, and the median age at diagnosis is 27 years. The registry classifies 75.9% as type 1 HAE, 20.0% as type 2, 2.7% as HAE-nC1-INH, and 1.4% as acquired angioedema. The median diagnostic delay was longest in type 2 HAE at 15 years (IQR: 2–25), followed by type 1 HAE at 11 years (IQR: 3–22). A positive family history is reported in 79.6% of cases, while 24.9% have consanguineous parents. Laryngeal attacks occurred in 38.4% of cases, highlighting disease severity. Misdiagnosis was observed in 52.8% of patients, with allergic angioedema (39.2%) and familial Mediterranean fever (20.08%) being the most common errors. A total of 15.6% of patients underwent unnecessary surgical procedures due to initial misdiagnosis.

**Conclusion:** The HAE/AAE-TR Registry provides valuable epidemiological and clinical data on hereditary and acquired angioedema in Turkey. The findings emphasize the need for increased disease awareness, earlier diagnosis, and improved patient management strategies. This structured, nationwide registry serves as a critical tool for enhancing patient care, standardizing diagnostic approaches, and contributing to global research on hereditary angioedema.

## O-44

### Case report: A 5-year-old boy with a truncating ANGPT1 mutation, possibly associated with hereditary angioedema with normal C1-inhibitor

Christina Weber-Chrysochoou^1^, S. Palmer Sarrott^2^, J.M. Galli^3^, J. Trück^4^

^1^Allergy Unit, Dermatology Department, University Hospital of Zurich, Zurich, Switzerland; ^2^Rheumatology Department, Children’s University Hospital of Zurich, Zurich, Switzerland; ^3^Ear Nose Throat Department, Children’s University Hospital of Zurich, Zurich, Switzerland; ^4^Division of Allergy and Children’s Research Center, University Children’s Hospital Zurich, University of Zurich, Zurich, Switzerland

Allergy, Asthma & Clinical Immunology 2026, 22(Suppl 1):O-44

**Background:** Hereditary angioedema (HAE) is a rare genetic disorder characterized by recurrent episodes of subcutaneous or submucosal swelling. While most cases are due to mutations in the SERPING1 or F12 genes, leading to C1-inhibitor (C1-INH) deficiency or dysfunction, a subset of patients presents with normal C1-INH levels and function. Recent studies have identified mutations in the ANGPT1 gene, encoding angiopoietin-1, as a cause of HAE with normal C1-INH.

**Case description:** We report a 5-year-old boy presenting with chronic recurrent pharyngeal swelling, described as "edematous" upon otolaryngological examination. A biopsy of the epiglottis ruled out malignancy. The patient also experienced recurrent abdominal pain, previously attributed to constipation, which did not respond to high-dose antihistamines or plasma-derived C1-INH (20 IU/kg body weight).

Additionally, the patient had a history of chronic recurrent arthralgia, leading to treatment with steroids, methotrexate, and adalimumab; however, therapy was discontinued due to lack of effect. Comprehensive imaging, including full-body and targeted hip MRI, was largely unremarkable. Notably, there was an absence of arthritis, but the patient experienced prolonged episodes of limping and pain, prompting initial immunosuppressive therapy.

Genetic testing revealed a heterozygous truncating mutation in ANGPT1 (p.Leu377Glnfs19*), inherited from the father, absent in population databases (gnomAD), and predicted to result in a non-functional protein. The family history was significant for systemic arthritis in both parents, with the father also reporting recurrent abdominal pain during his youth.

**Conclusion:** This case highlights a 5-year-old boy with a truncating *ANGPT1* mutation, presenting with clinical features consistent with HAE with normal C1-INH. The association between *ANGPT1* mutations and HAE with normal C1-INH has been previously documented. Our findings support the role of *ANGPT1* mutations in the pathogenesis of this HAE subtype. Further functional studies are warranted to elucidate the mechanisms by which ANGPT1 deficiency contributes to angioedema phenotypes and how to manage these patients. Consent to publish had been obtained.

## O-45

### Clinical evidence for a cross-talk between Mast-Cells and bradykinin-mediated angio-edema: Case report and discussion

Avner Reshef^1^,*, Iris Leibovich Nassi^1,2^

^1^Angioedema Research Center, Barzilai University Medical Center, Ashkelon, Israel; ^2^The Academic School of Nursing, Barzilai University Medical Center, Ashkelon, Israel.

*aresh@netvision.net.il

Allergy, Asthma & Clinical Immunology 2026, 22(Suppl 1):O-45

**Background:** The underlying canonical pathomechanism of Hereditary angioedema (HAE) due to C1-inhibitor deficiency (HAE-C1-INH), and some variants with normal C1-INH (HAE-nC1-INH), is vascular endothelial (VE) hyperpermeability owing to Bradykinin (BK) or intrinsic VE dysfunction (HAE-VE). In contrast, Urticaria (URT) is considered a distinct entity, manifested by wheals and erythema (hives), accompanied by intense pruritus, and mediated by activated mast-cell (MC) mediators. So far, few cases of URT have been reported among HAE patients, most of which are temporary or relapsing. Interactions between MC mediators and BK-mediated mechanisms have been demonstrated in the rodent model [1], antihistamines were shown to inhibit BK-induced cutaneous wheal-and-flare [2], and BK analog (Icatibant) released histamine from isolated skin MCs and elicited skin reactions attenuated by antihistamines [3]. MC's degranulation can release Heparin and other negatively charged polyanions capable of triggering the contact cascade [4]. The role of the MC MRGPRX2 receptor was also proposed as a putative linkage between the two systems [5]. Recently, two additional genetic variants of HAE-nC1-INH were described as co-segregating in families in which many patients had URT alongside angioedema, also implying a pathophysiologic link [6,7]

**Case report:** An 18-year-old female with an established diagnosis and family history of HAE-C1-INH-Type 1 had from early childhood typical URT attacks with intense pruritus and flushing. Her mother, diagnosed with the same endotype, is occasionally having hives as a prodrome. Some exacerbations of URT in the patient are accompanied by acral cyanosis involving the fingers and lower extremities, resembling the Raynaud Phenomenon. Laboratory investigation revealed antigenic C1-INH = 6.0 mg/dl, C4 < 2.9 mg/dl, positive anti-nuclear antibodies (1:320, nucleolar pattern) but negative dsDNA antibodies and absence of nuclear or nucleolar specific autoimmune markers. Hematology, coagulation, biochemistry, and immunology panels are normal. Baseline serum Tryptase was normal at (6.6 mcg/L). Attempted therapies included: C1-INH concentrate, Icatibant, antihistamines, Montelukast, and corticosteroids. Long-term prophylaxis was proposed but rejected by the family.

**Conclusion:** This case further supports the coexistence of the bradykinin-forming system and MC-mediated pathologies. Plausible cross-talk between the two phenotypically distinct entities will be discussed vis-a-vis the most recent published data. Consent to publish had been obtained.

References:

[1] Oschatz C, et al. Immunity 2011;34:258-268

[2] Fadel R, et al. Allergy 2000; 55(4):382-5

[3] Maurer M, Church MK. Exp Dermatol 2012;21(2):154-5 

[4] Hofman Z, et al., Clinic Rev Allergy Immunol 2016;51:152–161

[5] Porebski G, et al. Front Immunol 2024;15:1399459

[6] Vincent D, et al. J Allergy Clin Immunol Glob. 2024;3(2):100223

[7] D'Apolito M, et al. J Allergy Clin Immunol. 2024;154(3):698-706

## About this supplement

These abstracts have been published as part of Allergy, Asthma & Clinical Immunology Volume 22 Suppl 1, 2026. The full contents of the supplement are available online at https://doi.org/10.1186/s13223-026-01017-1. Please note that this is part 2 of 2.

